# CRISPR-Cas9 Targeting of Hepatitis B Virus Covalently Closed Circular DNA Generates Transcriptionally Active Episomal Variants

**DOI:** 10.1128/mbio.02888-21

**Published:** 2022-04-07

**Authors:** Maria Guadalupe Martinez, Emmanuel Combe, Aurore Inchauspe, Philippe Emmanuel Mangeot, Elodie Delberghe, Fleur Chapus, Gregory Neveu, Antoine Alam, Kara Carter, Barbara Testoni, Fabien Zoulim

**Affiliations:** a INSERM U1052, CNRS UMR-5286, Cancer Research Center of Lyon, Lyon, France; b Evotec, Lyon, France; c Centre International de Recherche en Infectiologie, INSERM U1111, CNRS UMR-5308, INSERM and Ecole Normale Superieure de Lyon, Lyon, France; d University of Lyon, Université Claude-Bernard, Lyon, France; e Hospices Civils de Lyon, Lyon, France; The Penn State University College of Medicine; Rutgers-Robert Wood Johnson Medical School

**Keywords:** hepatitis B virus, covalently closed circular DNA, CRISPR-Cas9

## Abstract

Chronic hepatitis B virus (HBV) infection persists due to the lack of therapies that effectively target the HBV covalently closed circular DNA (cccDNA). We used HBV-specific guide RNAs (gRNAs) and CRISPR-Cas9 and determined the fate of cccDNA after gene editing. We set up a ribonucleoprotein (RNP) delivery system in HBV-infected HepG2-NTCP cells. HBV parameters after Cas9 editing were analyzed. Southern blot (SB) analysis and DNA/RNA sequencing (DNA/RNA-seq) were performed to determine the consequences of cccDNA editing and transcriptional activity of mutated cccDNA. Treatment of infected cells with HBV-specific gRNAs showed that CRISPR-Cas9 can efficiently affect HBV replication. The appearance of episomal HBV DNA variants after dual gRNA treatment was observed by PCR, SB analysis, and DNA/RNA-seq. These transcriptionally active variants are the products of simultaneous Cas9-induced double-strand breaks in two target sites, followed by repair and religation of both short and long fragments. Following suppression of HBV DNA replicative intermediates by nucleoside analogs, mutations and formation of smaller transcriptionally active HBV variants were still observed, suggesting that established cccDNA is accessible to CRISPR-Cas9 editing. Targeting HBV DNA with CRISPR-Cas9 leads to cleavage followed by appearance of episomal HBV DNA variants. Effects induced by Cas9 were sustainable after RNP degradation/loss of detection, suggesting permanent changes in the HBV genome instead of transient effects due to transcriptional interference.

## INTRODUCTION

Despite the availability of a prophylactic vaccine, hepatitis B virus (HBV) remains a major public health concern (1). The HBV genome persists in the nucleus of infected hepatocytes as a 3.2-kb double-stranded episomal DNA known as covalently closed circular DNA (cccDNA). cccDNA is originated from the relaxed circular DNA (rcDNA) produced in the viral nucleocapsids during infection (2). While cccDNA is the only template for viral genomic DNA replication, both cccDNA and integrated HBV DNA can act as templates for subgenomic transcripts (3).

Current antiviral treatments, including nucleos(t)ide analogues (NAs) and pegylated alpha interferon (PEG-IFN), slow the progression of HBV-induced disease. However, they do not lead to complete viral elimination due to their inability to affect cccDNA (4, 5). The combination of available antiviral agents with direct cccDNA targeting could lead to a chronic hepatitis B (CHB) cure; therefore, finding strategies to directly target cccDNA remains essential (6). This goal has become feasible with designer nucleases such as bacterial CRISPR (clustered regularly interspaced short palindromic repeats)-Cas adapted for use in mammalian cells (7–9). The CRISPR-Cas9 system is easily reprogrammable, and different DNA sequences can be targeted by redesigning the guide RNAs (gRNAs) (10). Though several studies evaluated the potential of gene-editing approaches to target the HBV genome, the fate of the edited cccDNA molecules and their biological functions have not been evaluated (11, 12). Furthermore, the possibility that Cas9 could target HBV DNA replicative intermediates exhibiting the target sequence as double-stranded DNA (dsDNA), such as protein-free rcDNA, rather than cccDNA directly or before cccDNA establishment, has not been excluded. Altogether, an analysis of direct cccDNA targeting and its fate after CRISPR-Cas9 editing in a natural *de novo* infection model is still needed.

In this study, a gRNA/Cas9 complex was delivered using ribonucleoproteins (RNP) in HepG2-NTCP cells after infection establishment (13). CRISPR-Cas9 directly targeted cccDNA, leading to indel (insertion-deletion) formation. Southern blotting (SB), on-target DNA sequencing, and transcriptome sequencing (RNA-seq) showed that dual gRNAs treatments led formation of novel HBV DNA variants that remained transcriptionally active.

## RESULTS

### Screening for efficient target sites in the HBV genome.

**FIG 1:**
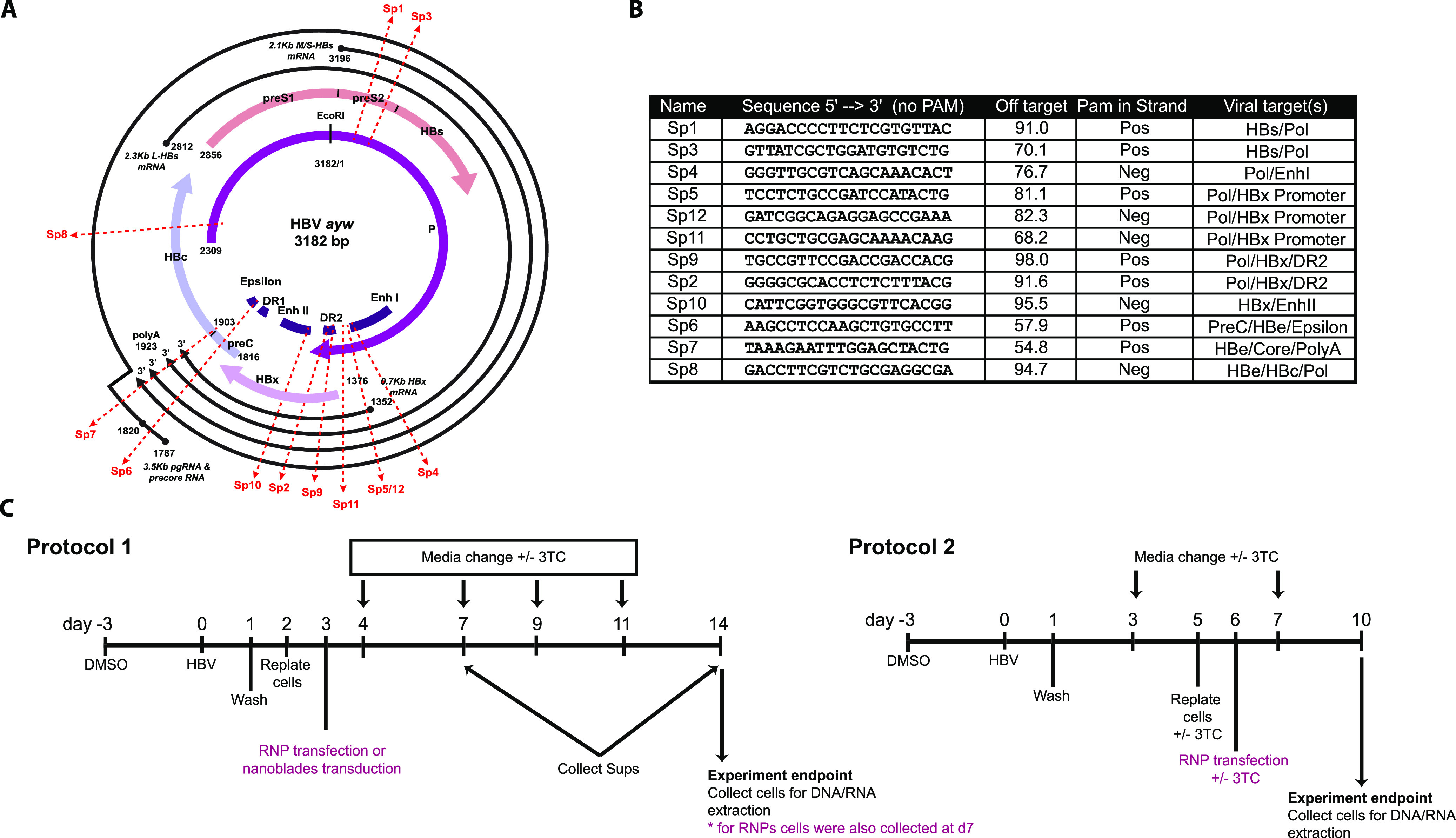
Overview of specific S.py Cas9 gRNAs and protocols to target the HBV genome. (A) Schematic representation of the HBV genome with the unique EcoRI restriction site at the top, showing the location of the gRNA-targeted sequences (genotype D, *ayw* strain), The predicted viral transcription start sites and regulatory regions (enhancers I and II) are indicated. The labels Sp1 to -12 mark the gRNA target sites. (B) gRNA sequences and potential target sites. Off-target effects predicted by the Benchling platform are indicated for each gRNA. (C) Experimental schematic for the protocol timeline used in this work.

**FIG 2:**
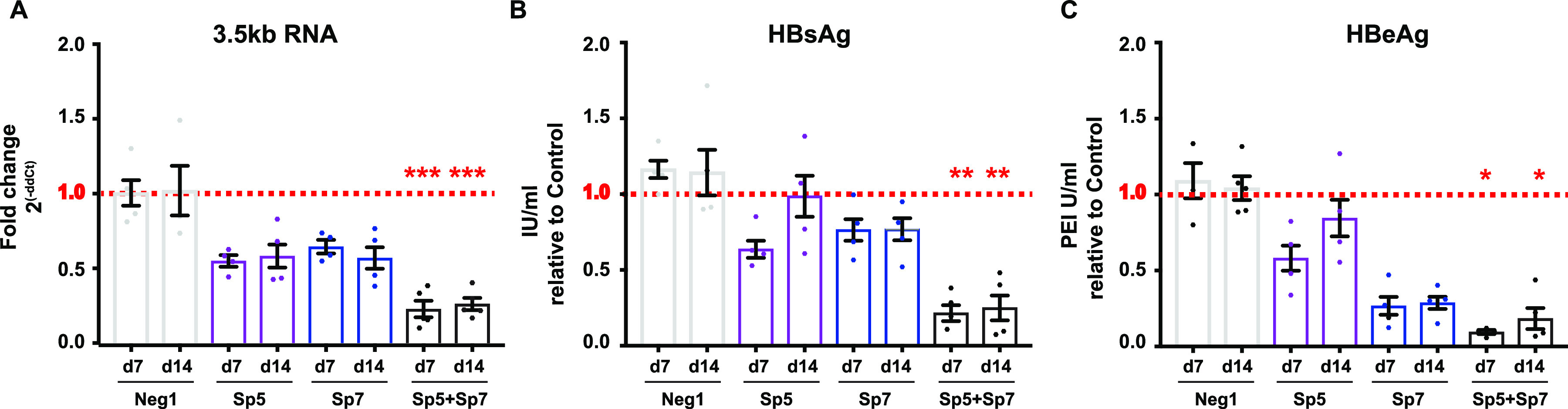
Effects of Sp5 and Sp7 in HepG2-NTCP cells infected *de novo* with HBV endure after Cas9 protein is degraded. Cells were infected following protocol 1 in Fig. 1C and treated with gRNA/Cas9. (A) Total RNA was extracted from cell lysates at 7 or 14 dpi, and 3.5-kb RNA was measured by quantitative reverse transcription-PCR (qRT-PCR). (B) HBsAg and (C) HBeAg concentrations in supernatant were collected at 7 or 14 dpi and measured by ELISA. HBV-only samples were normalized to 1 (red dotted lines). Error bars indicate standard errors (SE) for ≥4 replicates. Statistical differences relative to control were analyzed by nonparametric analysis of variance (ANOVA) (*, *P* < 0.05; **, *P* < 0.01; ***, *P* < 0.001).

gRNAs targeting different HBV genomic regions, either individually or in combinations, were evaluated for their efficiency to reduce HBV replication (Fig. 1A and B). A protocol was optimized to deliver Cas9 RNPs in infected HepG2-NTCP cells and study the effect of CRISPR-Cas9 in *de novo* established cccDNA (protocol 1) (Fig. 1C and data not shown). Levels of HBV e antigen (HBeAg) and HBV surface antigen (HBsAg) were measured in supernatants, while those of a 3.5-kb RNA were measured intracellularly. A consistent reduction in viral parameters compared to infected untreated samples (normalized to 1, indicated by the dotted red line in the graphs shown in Fig. S1A-C and S2D-F) or to negative-control conditions (Neg1, Neg2, or nonessential gene for cell homeostasis [HPRT]) was observed for several single and dual gRNA combinations at 7 days postinfection (dpi) (Fig. S1 and S2). Given their location in critical regulatory regions of the HBV genome, Sp5, Sp7, and their combination were chosen for a deeper analysis (Fig. 1). Time course experiments demonstrated that the combination of Sp5 and Sp7 led to reduction in 3.5-kb-RNA levels and release of HBe and HBs antigens. These effects were sustained at 14 dpi (Fig. 2).

10.1128/mBio.02888-21.2 FIG S1 Small-scale screening of single- or dual-gRNA treatment in HepG2-NTCP cells infected *de novo* with HBV. Cells were infected and RNP transfected following protocol 1 in Fig. 1C. (A) Total RNA was extracted from cell lysates, and 3.5-kb RNA was measured by qRT-PCR. (B) HBsAg and (C) HBeAg concentrations in supernatant were measured by ELISA. HBV-only samples were normalized to 1 in each case (dotted red lines). Neg1 and Neg2, nontargeting gRNAs; HPRT, gRNA targeting a cellular gene that does not affect HBV. Error bars indicate SE for 5 or 8 replicates. Statistical differences relative to control were analyzed by nonparametric ANOVA (*, *P* < 0.05; **, *P* < 0.01; ***, *P* < 0.001; ****, *P* < 0.0001). Download FIG S1, PDF file, 0.06 MB. Copyright © 2022 Martinez et al. 2022 Martinez et al. https://creativecommons.org/licenses/by/4.0/ This content is distributed under the terms of the Creative Commons Attribution 4.0 International license.

10.1128/mBio.02888-21.3 FIG S2 Sp5 and Sp7 effects in viral parameters are independent on their delivery and do not lead to transcriptional interference. HepG2-NTCP cells at 80 to 90% confluency were transduced with nanoblades carrying the Cas9 protein and no gRNA, gRNA targeting the cellular gene EMX1, Sp5, Sp7, or Sp5+Sp7. (A) Cells were fixed 24 h posttransduction and stained with anti-Cas9 antibody for epifluorescence microscopy. (B) Total DNA was extracted at 4 dpt, and PCR was performed as indicated in Materials and Methods using primers outside the Cas9 cleavage site for EMX1. PCR products were digested with T7E1 and separated in an agarose gel. The control was cells treated with nanoblades carrying only Cas9. As expected, EMX1 cleavage products after T7E1 digestion were observed only when crRNA targeting EMX1 was used. (C) Cells were infected, and RNPs were transduced with nanoblades following protocol 1 in Fig. 1C. Total DNA was extracted from cell lysates; then, PCRs were performed to determine the presence of HBV CRISPRv1 and CRISPRv2. (D) HBsAg and (E) HBeAg concentrations in supernatant collected at 7 or 14 dpi were measured by ELISA. (F) Cells were infected and RNP assembled with S.py dead Cas9 (dCas9) transfected following protocol 1 in Fig. 1C. Total RNA was extracted from cell lysates, and 3.5-kb RNA was measured by qRT-PCR. HBsAg and HBeAg concentrations in supernatant were measured by ELISA. HBV-only samples were normalized to 1 in each case (dotted red lines). Data were normalized to HBV-only samples. Error bars indicate SE of 4 replicates. Statistical differences relative to controls were analyzed by nonparametric ANOVA (*, *P* < 0.05; **, *P* < 0.01; ***, *P* < 0.001). Neg1 and Neg2, nontargeting gRNAs; HPRT, gRNA targeting a cellular gene that does not affect HBV. Download FIG S2, PDF file, 1.4 MB. Copyright © 2022 Martinez et al. 2022 Martinez et al. https://creativecommons.org/licenses/by/4.0/ This content is distributed under the terms of the Creative Commons Attribution 4.0 International license.

To discard any potential bias due to the RNP delivery strategy using lipid-based transfection, Sp5 and Sp7 RNPs were delivered using nanoblades (14). Similar reductions in viral protein production were observed, suggesting that effects of the gRNA-Cas9 combination are independent of the delivery strategy (Fig. S2D and E). To control for nuclease-independent effects, infected cells were transfected with RNPs assembled with a nuclease-deficient Cas9 (D10A/H840A; Streptococcus pyogenes [S.py] dCas9). The mild reduction in the viral parameters with the combination of Sp5 and Sp7/dCas9 at 7 dpi was reversed at 14 dpi, indicating a transient effect due to dCas9 binding to the viral DNA (Fig. S2F). This is likely the result of transcriptional interference due to the binding of the dCas9 protein to HBV DNA, leading to a transient alteration in the HBV cycle. This effect disappeared after the Cas9 protein was degraded, which for RNP transfection is around 3 days posttransduction (dpt) (data not shown) (15). In contrast, evaluation of viral parameters at 14 dpi (11 dpt) indicated that the effects of Sp5+Sp7 in combination with Cas9 were sustained after the RNP was degraded.

### Dual HBV genome targeting leads to the formation of novel viral episomes.

**FIG 3:**
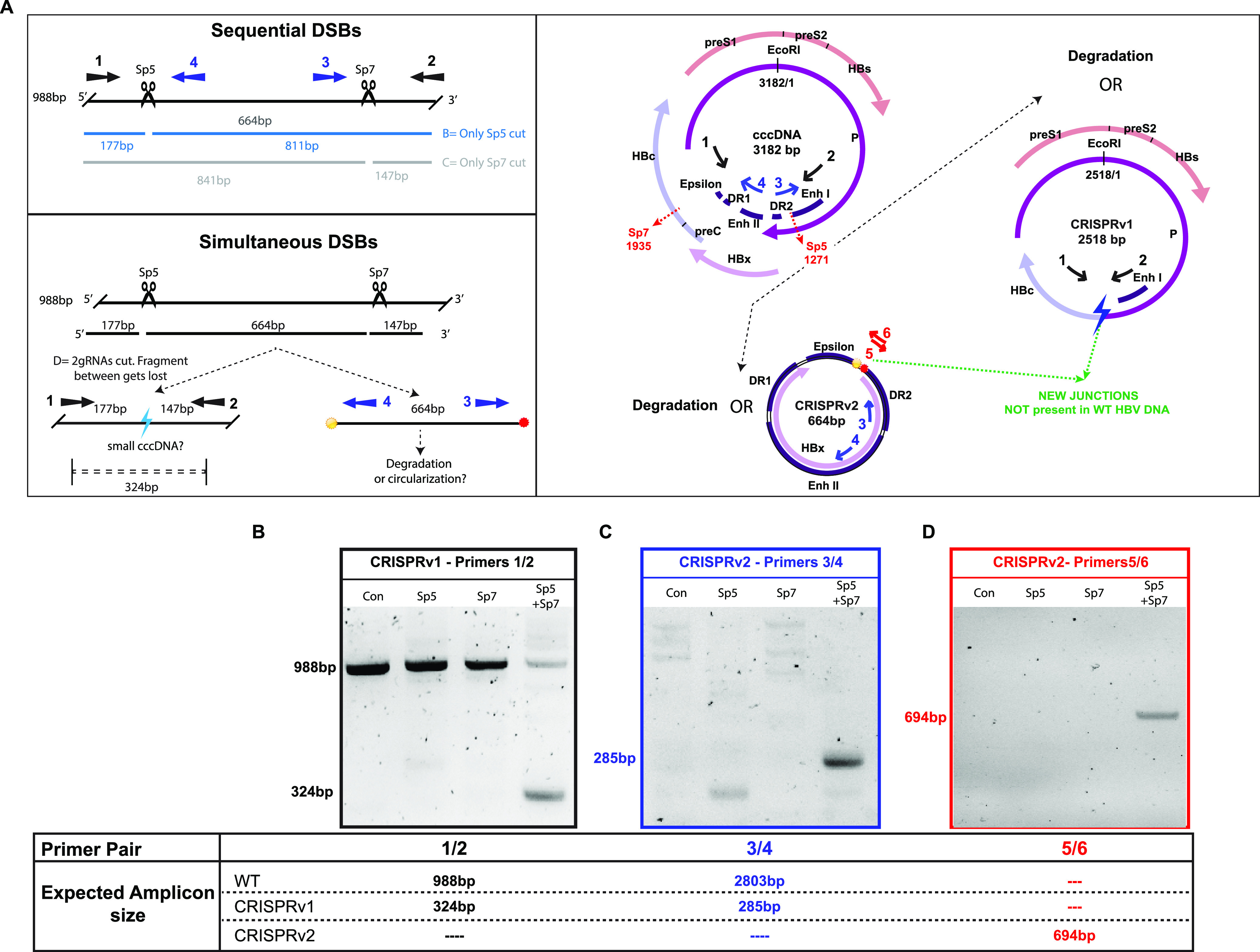
Formation of novel HBV DNA variants after dual gRNA targeting. (A) Schematic of potential outcomes after dual gRNA cleavage. (Top left) Expected indels after sequential Cas9 cleavage. Black arrows indicate primer locations for PCR amplification relative to the Cas9 cleavage site to detect full-length cccDNA (988 bp) or CRISPRv1 (325 bp) (primers 1 and 2). (Bottom left) Simultaneous Cas9 cleavage leading to the excision of the ∼664-bp fragment between the 2 target sites and religation generating novel variants: CRISPRv1, ∼2,516 bp (detected by primers 1 and 2 [black arrows]; 324 bp), and CRISPRv2, ∼664 bp (detected by PCR with primers 3 and 4 [blue arrows]; 285 bp). (Right) Schematic representation of different HBV DNA species after CRISPR-Cas9 dual targeting. (B to D) Cells were infected following protocol 1 (Fig. 1C). Cell lysates were collected, and cccDNA was purified by nuclear fractionation followed by modified Hirt extraction. (B) PCR was performed as indicated in Materials and Methods using primers flanking Sp5 and Sp7 target sites to detect the presence of CRISPRv1 (primers 1 and 2). (C) PCR using primers inside the excised region was used to detect the presence of CRISPRv2 (primers 3 and 4) and short elongation times to favor amplicons of <1 kb. (D) Primers with the exact expected junction after religation of the 664-bp fragment (CRISPRv2) were used to validate the presence of CRISPRv2 (primers 5 and 6). Amplicons were separated on an agarose gel. Expected sizes of PCR amplified cleavage products are indicated in the table.

Using dual gRNAs could lead to different outcomes depending on the efficiency and kinetics of double-strand breaks (DSBs) on target sites: (i) sequential DSBs leading to point mutations in both target sites (Fig. 3A, top); (ii) simultaneous DSBs leading to removal of the fragment between the two target sites (if the repair system can religate the extremities generated by the DSBs, smaller PCR products would be detected using specific primers sets [Fig. 3A, bottom, black and blue arrows]); (iii) an insufficient or delayed nonhomologous-end-joining (NHEJ) repair system not able to rejoin the abundant Cas9-induced DSBs, leading to cleaved viral template degradation. Samples enriched in cccDNA by nuclear fractionation followed by Hirt extraction were subjected to PCR amplification using primers flanking the target region (primers 1 and 2 [black arrows in Fig. 3A; also, see Fig. S8E]). When infected cells were treated with single gRNAs, an amplicon of expected full length was obtained (988 bp) (Fig. 3B). When both Sp5 and Sp7 were used, bands of different sizes were detected, with one particularly evident band with the expected size corresponding to the cleaved product (324 bp), referred to here as CRISPR variant s-1 (CRISPRv1) (Fig. 3). To investigate the outcome of the fragment between the target sites, 2 pairs of primers were designed: a pair flanking the predicted junction (primers 3 and 4 [Fig. 3A, blue arrows]) and a second pair with a sequence spanning the exact predicted junction after Cas9 cleavage in the position −3 from the protospacer-adjacent motif (PAM) (primers 5 and 6 [Fig. 3, red arrows]). A band of the expected PCR size corresponding to the circularization of the fragment between the Sp5 and Sp7 target sites was found when both primer pairs were used (Fig. 3C and D), suggesting that the fragment excised by dual Cas9 targeting was religated (CRISPR variant s-2 [CRISPRv2]). These results suggest that HBV DNA can be edited and repaired after CRISPR-Cas9 cleavage. Furthermore, excision of a fragment of around 664 bp containing the complete HBx open reading frame (ORF) and major regulatory regions (DR2, EnhII, DR1, polyadenylation signal, and epsilon) led to the formation of HBV DNA variants: CRISPRv1, which is around 2,518 bp and lacks major regulatory regions, and CRISPRv2, which is around 664 bp and is formed mainly by regulatory regions. Furthermore, formation of these variants was independent of the delivery strategy (Fig. S2C).

10.1128/mBio.02888-21.9 FIG S8 Formation of CRISPRv1 and CRISPRv2 occurs by direct cccDNA targeting after dual gRNA/Cas9 targeting, and these variants are transcriptionally active. Cells were infected following protocol 2 (Fig. 1C) in the presence or absence of 3TC prior to gRNA/Cas9 RNP transfection. Cell lysates were collected, and cccDNA was purified by nuclear fractionation followed by modified Hirt extraction. PCR was performed as indicated in Materials and Methods using (A) primers flanking Sp5 and Sp7 target sites to detect the presence of CRISPRv1 (primers 1 and 2; top panels), and (C) primers inside the excised region were used to detect the presence of CRISPRv2 (primers 3 and 4; bottom panels). Amplicons were separated on an agarose gel. Expected cleavage products and sizes are indicated on the left of the gel (A and C). (B and D) Amplicons were gel purified and sent for on-target amplicon sequencing. A custom reference genome with the expected target sites of Cas9 in the HBV genome followed by recircularization of the double-cleaved fragments was generated, and amplicon sequences were aligned to their respective variants using a padding of 20 using IGV. Under these conditions, it is clear that the bands of 324 bp (A) and 285 bp (C) correspond to the expected CRISPRv1 (B) and CRISPRv2 (D) and are present both in the presence or absence of 3TC, suggesting direct cccDNA targeting. (E) Primers used to generate amplicons for DNA amplicon sequencing and PCRs to detect CRISPRv1 and CRISPRv2. Detection was achieved with the PCR1 setup displayed in all figures. PCR2 is an additional PCR with extended primers to generate sufficient DNA material for amplicon sequencing. (F and G) RNA sequencing products from control samples or samples treated with Sp5+Sp7, in the presence or absence of 3TC, following protocol 2 were aligned to a custom reference genome generated with the expected target sites of Cas9 in the HBV genome followed by recircularization of the double-cleaved fragments, and amplicons were aligned to their respective variants using a padding of 18 using IGV. Under these conditions, it is clear that the expected CRISPRv1 and CRISPRv2 are transcriptionally active, as they are present in the unbiased RNA-seq approach only when the infected cells were treated with the Sp5+Sp7 combination. Colors in the alignments represent the following changes compared to the reference sequence: purple, insertions; blank spaces, deletions; red, any nucleotide → T; green, any nucleotide → A; orange, any nucleotide → G; blue, any nucleotide → C. Download FIG S8, PDF file, 0.4 MB. Copyright © 2022 Martinez et al. 2022 Martinez et al. https://creativecommons.org/licenses/by/4.0/ This content is distributed under the terms of the Creative Commons Attribution 4.0 International license.

**FIG 4:**
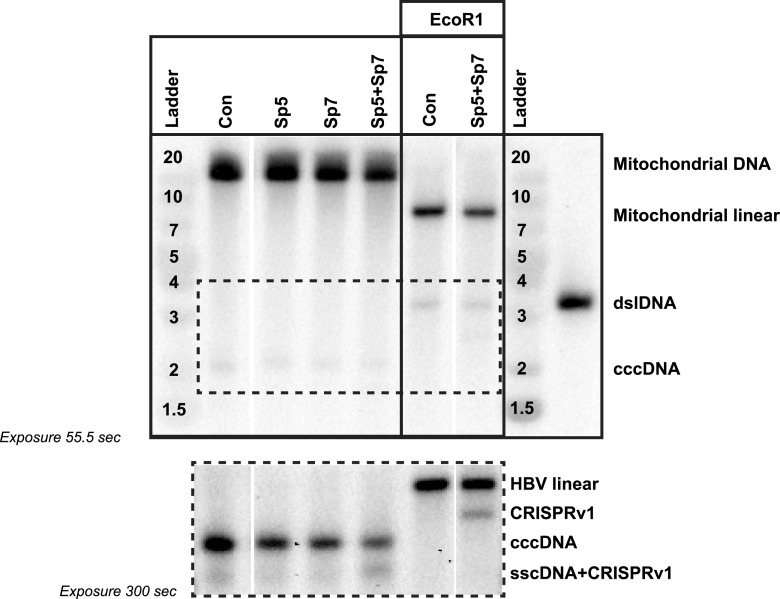
CRISPR-Cas9 cccDNA disruption and formation of smaller HBV variants. Intracellular cccDNA in total Hirt extractions followed by ExoI+III digestion from HBV-infected HepG2-NTCP cells following protocol 1 (Fig. 1C) was analyzed by SB using HBV negative-strand-specific probes or ND4 mitochondrial DNA probes. gRNA treatments are indicated at the top. The figure is representative of 4 independent experiments. (Right) Samples digested for linearization with EcoRI. (Bottom) Higher exposure (300 s) of the outlined area in the top panel.

To unequivocally determine the presence and episomal conformation of these CRISPR variants, we performed SB analysis from samples where episomal DNA was enriched by Hirt extraction followed by digestion with exonucleases I and III (ExoI+III). As previously described, due to the high temperatures used for exonuclease inactivation, an additional band corresponding to the single-stranded circular DNA (sscDNA) was detected (Fig. S3) (16). This band migrates at the expected size for the CRISPRv1 HBV (Fig. 4) but was eliminated after HBV DNA linearization with EcoRI (Fig. 4, EcoRI Con column). While the no-Cas9 control sample showed only the expected wild-type (WT) cccDNA band (3,182 bp), the Sp5+Sp7 combination led to one additional smaller band corresponding in size to the removal of the fragment between the two target sites (CRISPRv1; around 2,518 bp) (Fig. 4). CRISPRv2 was not detected given its smaller size and lack of probe sensitivity in this region. These data suggest that dual gRNA treatment leads to formation of novel episomal HBV DNA variants.

10.1128/mBio.02888-21.4 FIG S3 Formation of sscDNA in DNA treated with ExoI+III. Intracellular cccDNA in total Hirt extractions from HBV infected HepG2-NTCP cells was analyzed by Southern blotting using HBV negative-strand-specific probes. Samples were processed differently to examine the formation of the smaller band detected by the HBV probes. ExoI+III, digestion for 2 h at 37°C; T5, digestion for 2 h at 37°C and for 5 min at 95°C before running the samples. Different treatment combinations are indicated below the gel. The sscDNA band is visible in lane 2 (ExoI+III digestion only) and more evident in lane 3, after heat denaturation. dp-rcDNA, deproteinized relaxed circular DNA; dslDNA, double-strand linear DNA. Download FIG S3, PDF file, 0.3 MB. Copyright © 2022 Martinez et al. 2022 Martinez et al. https://creativecommons.org/licenses/by/4.0/ This content is distributed under the terms of the Creative Commons Attribution 4.0 International license.

### Spectrum of cccDNA mutations induced by CRISPR-Cas9.

**FIG 5:**
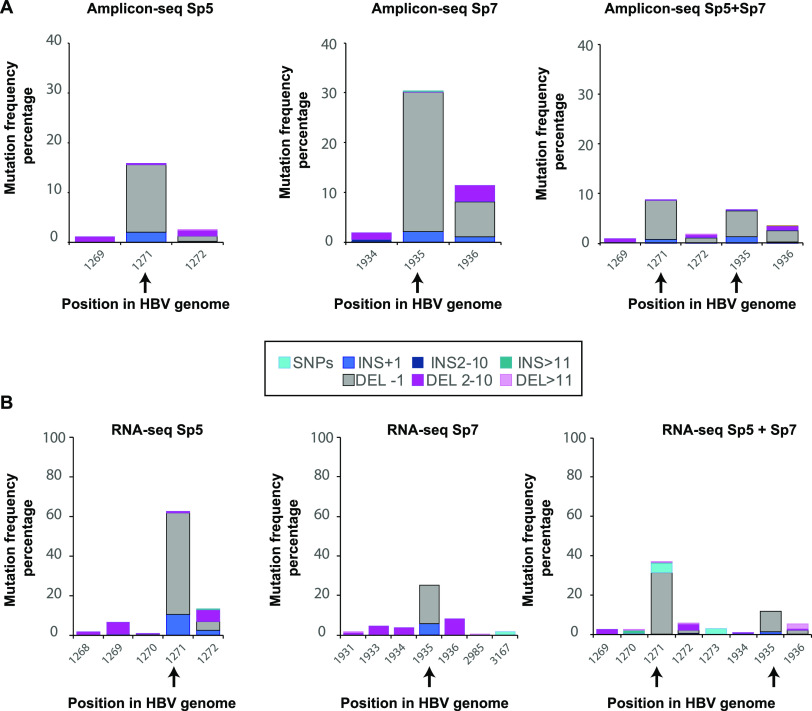
Mutational and transcriptional profile of cccDNA variants after CRISPR-Cas9 cleavage. Cells were infected following protocol 1 (Fig. 1C) and transfected with RNPs assembled with gRNA/Cas9. (A) Amplicons were generated from cccDNA-enriched samples, by nuclear fractionation, followed by Hirt extraction and ExoI+III digestion, using primers flanking the gRNA target regions, and sent to Genewiz for on-target amplicon sequencing. (B) Total RNA was extracted and processed for cDNA library preparation with rRNA depletion for Illumina sequencing. Data are percentages of mutant reads relative to the HBV reference genome. In both cases, all positions in the HBV genome with mutations present at 1% or more are displayed. Mutations were categorized as single nucleotide insertions or deletions, insertions or deletions of 2 to 10 nucleotides (nt), or insertions or deletions of >11 nt, indicated by different colors in the bars for certain HBV DNA positions.

The spectrum of cccDNA mutations after CRISPR-Cas9 cleavage was evaluated by on-target amplicon sequencing on cccDNA-enriched samples obtained by nuclear fractionation, followed by Hirt extraction and ExoI+III digestions (further detailed in Materials and Methods). Amplicons generated as shown in Fig. 3B and C were gel purified for on-target amplicon sequencing (Genewiz). When treated with single gRNAs, both Sp5 and Sp7 consistently led to the expected patterns of Cas9-induced mutations (17), with a single nucleotide deletion in the expected Cas9 target site at position −3 from the PAM being the most frequently found mutation (Fig. 5A; positions with mutations present at 1% or more are displayed). When treated with the Sp5+Sp7 combination, the presence of single nucleotide deletions in the expected target site was evident, suggesting that the DSBs induced by different gRNAs occurred in different molecules or sequentially in the same molecule.

In accordance with PCR data suggesting the existence of CRISPRv1 (presumably lacking major known regulatory elements) and CRISPRv2 (exclusively formed by these regulatory regions and the complete HBx ORF), amplicon sequencing from the bands corresponding to these variants (Fig. 3B and C, Sp5 and Sp7) detected reads that correspond to these specific junctions (Fig. S4). To evaluate the transcriptional activity of the variants formed as a consequence of Cas9 cleavage, we performed RNA-seq analysis in infected HepG2-NTCP cells treated with Sp5 or Sp7 individually or in combination (Fig. 5B). Unbiased RNA sequencing indicated that the species found in HBV DNA were transcriptionally active, further confirming that the mutations are present in cccDNA, instead of other replicative intermediates. No off-target mutations (Fig. 5B [positions with mutations present at 1% or more are displayed]; Fig. S5A [positions with mutations present at 0.001% or more are displayed]) were observed in the HBV transcriptome outside the Cas9 target sites for both gRNAs. Additionally, off-target effects were evaluated *in silico* using Cas-OFFinder under low-stringency conditions (mismatch < 6, DNA bulge < 2, and RNA bulge < 2) (18): no other targets were predicted in the HBV genome genotype *ayw* (data not shown). Reads that correspond to the RNA transcribed from the CRISPRv1 and CRISPRv2 were found only in the Sp5+Sp7-treated samples when aligned to their custom reference genomes, indicating that they are transcriptionally active (Fig. S5B and C). Furthermore, Sp5 and Sp7 led to single nucleotide deletions translating to an early stop codon in the Pol (nucleotide position 1284) and HBe/HBc (nucleotide position 1992) ORFs, respectively (see supplemental results in Text S1 for details). This resulted in reduced intracellular HBc and HBs protein expression (Fig. S6).

10.1128/mBio.02888-21.1 TEXT S1 Supplemental materials and results. Download Text S1, DOCX file, 0.1 MB. Copyright © 2022 Martinez et al. 2022 Martinez et al. https://creativecommons.org/licenses/by/4.0/ This content is distributed under the terms of the Creative Commons Attribution 4.0 International license.

10.1128/mBio.02888-21.5 FIG S4 On-target amplicon sequencing of CRISPRv1 and CRISPRv2. Amplicons generated as shown in Fig. 3B and C were gel purified and sent for on-target amplicon-sequencing. Two custom reference genomes with the expected result of target site cleavage of Cas9 in the HBV genome followed by repair and religation of both short and long fragments were generated, and amplicon sequences were aligned using Integrative Genomics Viewer (IGV) to their respective variants using a padding of 20. Under these conditions, it is clear that the bands presented in Fig. 3B and C correspond to the expected CRISPRv1 (A) and CRISPRv2 (B). Colors in the alignment represent the following changes compared to the reference sequence: purple, insertions; blank spaces, deletions; red, any nucleotide → T; green, any nucleotide → A; orange, any nucleotide → G; blue, any nucleotide → C. Download FIG S4, PDF file, 0.1 MB. Copyright © 2022 Martinez et al. 2022 Martinez et al. https://creativecommons.org/licenses/by/4.0/ This content is distributed under the terms of the Creative Commons Attribution 4.0 International license.

10.1128/mBio.02888-21.6 FIG S5 Transcriptional profile of cccDNA after CRISPR-Cas9 cleavage featuring all mutations present with over 0.001% frequency and CRISPR variants. (A) RNA-seq data set presented in Fig. 5B showing all mutations present in the HBV transcriptome with a frequency of over 0.001% (in Fig. 5 and for display purposes, only frequencies over 1% are shown). Mutations present at 0.001% or more are displayed as the ratio of mutant reads to the sum of mutated or unmutated reads aligning with the same HBV reference genome region. Mutations found were categorized as single nucleotide insertions or deletions, insertions or deletions of 2 to 10 nt, or insertions or deletions of >11 nt, indicated by different colors in the bars at certain HBV DNA positions. (B and C) Control samples or samples treated with Sp5+Sp7 were aligned to a custom HBV reference genome sequence. The reference HBV sequence was generated to match the expected target sites of Cas9 followed by recircularization of the double-cleaved fragments. Amplicons were aligned to its respective variant using a padding of 18 using IGV. Under these conditions, it is clear that the expected CRISPRv1 and CRISPRv2 are transcriptionally active, as they are present in the unbiased RNA-seq approach only when the infected cells were treated with the Sp5+Sp7 combination. Colors in the alignment represent the following changes compared to the reference sequence: purple, insertions; blank spaces, deletions; red, any nucleotide → T; green, any nucleotide → A; orange, any nucleotide → G; blue, any nucleotide → C. Download FIG S5, PDF file, 0.2 MB. Copyright © 2022 Martinez et al. 2022 Martinez et al. https://creativecommons.org/licenses/by/4.0/ This content is distributed under the terms of the Creative Commons Attribution 4.0 International license.

10.1128/mBio.02888-21.7 FIG S6 HBc and HBs protein reduction after CRISPR-Cas9 cleavage. Cells were infected with HBV 3 days prior to the transfection with the RNPs complex using WT Cas9 targeting single or dual HBV regions, as indicated on the left, following the protocol 1 timeline in Fig. 1C. Cells were fixed at 7 dpi and processed for immunofluorescence against HBc (left) or HBs (right). Download FIG S6, PDF file, 0.1 MB. Copyright © 2022 Martinez et al. 2022 Martinez et al. https://creativecommons.org/licenses/by/4.0/ This content is distributed under the terms of the Creative Commons Attribution 4.0 International license.

### CRISPR-Cas9 cccDNA targeting in NA-treated cells.

**FIG 6:**
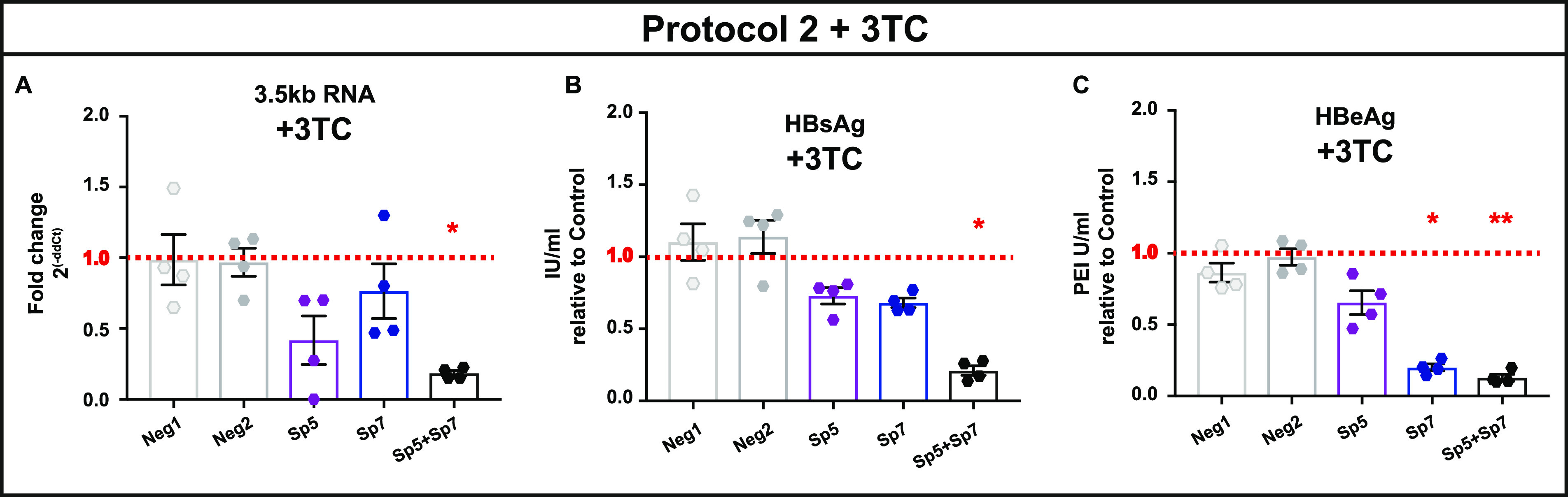
Direct effect of CRISPR-Cas9 in cccDNA cleavage. Cells were infected with HBV, and 3TC was added 4 days later, following protocol 2 (Fig. 1C). (A) Total RNA was extracted from cell lysates at 11 dpi, and 3.5-kb RNA was measured by qRT-PCR. (B) HBsAg and (C) HBeAg concentrations in supernatant were measured by ELISA. HBV-only samples in the presence of 3TC were normalized to 1 (red dotted lines). Error bars indicate SE for 4 replicates. Statistical differences relative to control were analyzed by nonparametric ANOVA (*, *P* < 0.05; **, *P* < 0.01).

Given the sequence similarity between HBV DNA intermediates (e.g., rcDNA, protein free (PF)-rcDNA, and cccDNA), gRNA/Cas9 complexes could target any of these intermediates that include the gRNA target sequence as dsDNA. To investigate the direct impact of gRNA/Cas9 on cccDNA, infected cells were pretreated with 2′,3′-didéoxy-3′-thiacytidine (3TC) to reduce viral DNA replicative intermediates (Fig. S7A), enriching the HBV genomic species in cccDNA before gRNA/Cas9 treatment (Fig. 1C, protocol 2). Similarly to protocol 1, results from this different timeline showed a reduction of the viral parameters following gRNA treatment, consistent with the results obtained in the absence of 3TC (Fig. S7B). In the continuous presence of 3TC, treatment with Sp5+Sp7 led to a reduction of 3.5-kb RNA, HBeAg, and HBsAg compared to untreated condition (normalized to 1; Fig. 6, red dotted lines) or samples treated with 2 nontargeting gRNAs (Neg1 and Neg2) (Fig. 6). These results suggest that gRNA/Cas9 can directly target cccDNA in the context of reduced replicative intermediates.

10.1128/mBio.02888-21.8 FIG S7 Effect of selected gRNAs in protocol 2 timeline in the absence of 3TC. (A) Effect of 3TC in HBV. Cells were infected with HBV and 3TC was added, or not, 4 days later. Total DNA was extracted from cell lysates at 11 dpi. Intracellular total HBV DNA was quantified by qPCR. For cccDNA quantification by qPCR, incomplete double-stranded circular DNA was degraded by ExoI+III treatment as indicated in Materials and Methods. Primers targeting a specific region in cccDNA were used. In both cases, an HBV standard curve was used to obtain absolute values of total DNA copies or cccDNA. The HBB reference gene was used to estimate the number of copies per nucleus. Total RNA was extracted from cells lysates at 11 dpi, and intracellular 3.5-kb RNA was measured by qRT-PCR. The 3.5-kb RNA was quantified relative to the GUSB reference gene using the 2^ΔΔ^*^CT^* method. HBsAg and HBeAg concentrations in supernatant were measured at the indicated time points by ELISA. All measurements were performed after 3 days of accumulation on medium. Data are normalized to HBV-only samples in the absence of 3TC to validate the expected effect of this treatment. (B) Cells were infected with HBV following protocol 2 in the absence of 3TC (Fig. 1C). Total RNA was extracted from cell lysates at 11 dpi, and 3.5-kb RNA was measured by qRT-PCR. HBsAg and HBeAg concentrations in supernatant were measured by ELISA. HBV-only samples in the absence of 3TC were normalized to 1 in each case (dotted red lines). Error bars indicate SE for 4 replicates. Statistical differences relative to the control were analyzed by nonparametric ANOVA (*, *P* < 0.05; **, *P* < 0.01; ****, *P* < 0.0001). (C) Cells were infected with HBV following protocol 2 in the absence of 3TC (Fig. 1C). cccDNA was extracted as detailed in Materials and Methods by nuclear extraction and modified Hirt purification followed by ExoI+III digestion. Amplicons were generated using nested primers flanking the gRNA target regions (Fig. S8E) and were sent to Genewiz to be processed for MiSeq amplicon sequencing. Mutations present at 1% or more are displayed. Mutations found were categorized as single nucleotide insertions or deletions, insertions or deletions of 2 to 10 nt, or insertions or deletions of >11 nt, indicated by different colors in the bars for certain HBV DNA positions. (D) Cells were infected with HBV following protocol 2 in the absence of 3TC (Fig. 1C). Total RNA was then extracted and processed for cDNA library preparation with rRNA depletion for Illumina sequencing. Mutations present at 1% or more are displayed. Mutations found were categorized as single nucleotide insertions or deletions, insertions or deletions of 2 to 10 nt, or insertions or deletions of >11 nt, indicated by different colors in the bars for certain HBV DNA position. Download FIG S7, PDF file, 0.06 MB. Copyright © 2022 Martinez et al. 2022 Martinez et al. https://creativecommons.org/licenses/by/4.0/ This content is distributed under the terms of the Creative Commons Attribution 4.0 International license.

**FIG 7:**
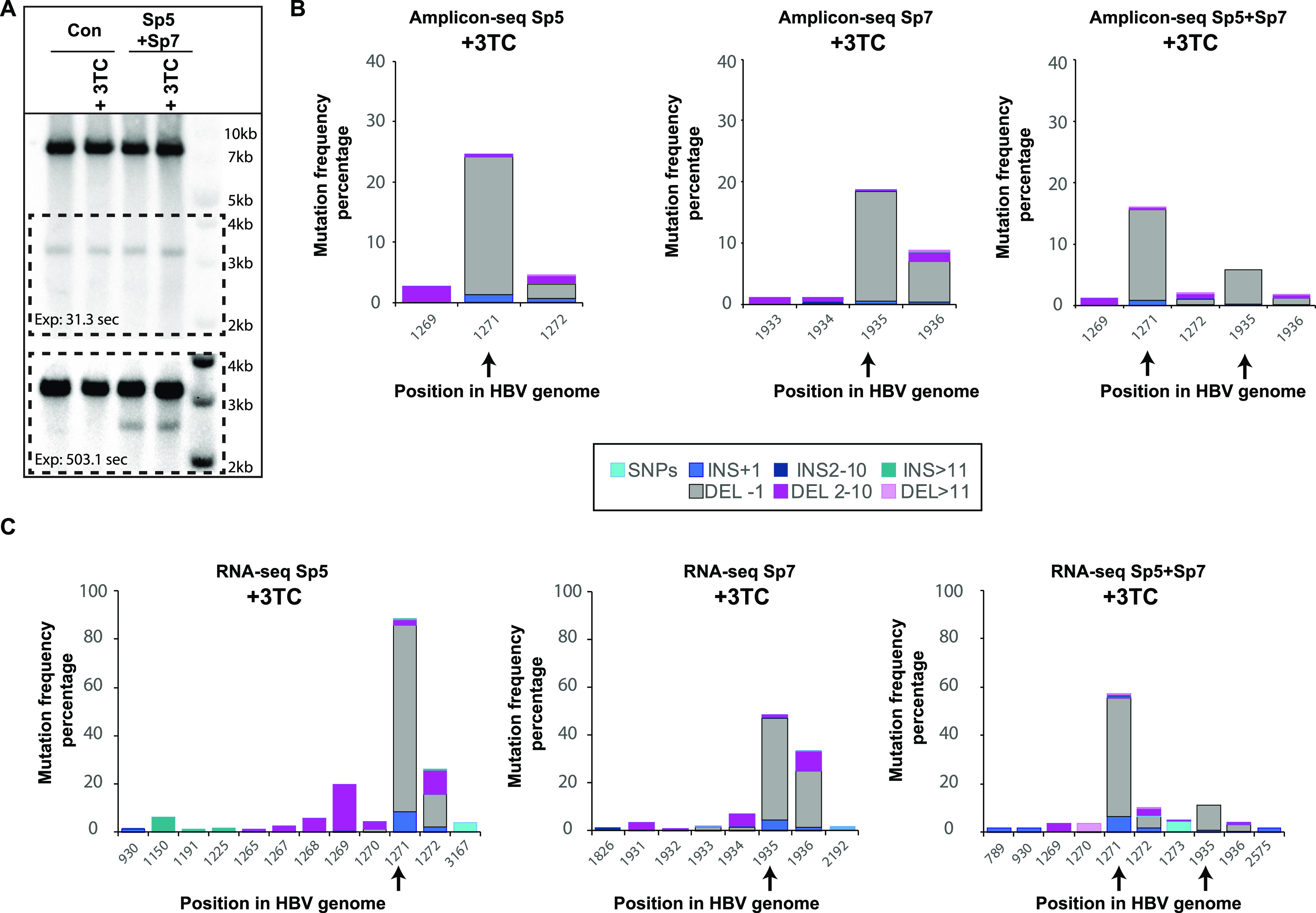
Mutational profile and transcriptional activity of novel HBV variants after CRISPR-Cas9 cleavage in the presence of 3TC. Cells were infected with HBV, and 3TC was added 4 days later following protocol 2 (Fig. 1C). Intracellular cccDNA was digested for linearization with EcoRI and analyzed by SB (A). gRNA and 3TC treatments are indicated at the top. (Bottom) Higher exposure of the outlined area in the top panel. (B) Amplicons were generated from cccDNA-enriched samples using primers flanking the gRNA target regions and sent to Genewiz for on-target amplicon sequencing. (C) Total RNA was extracted and processed for cDNA library preparation with rRNA depletion for Illumina sequencing. Data are percentages of mutant reads relative to the HBV reference genome. In both cases, all positions in the HBV genome with mutations present at 1% or more are displayed. Mutations were categorized as single nucleotide insertions or deletions, insertions or deletions of 2 to 10 nt, or insertions or deletions of >11 nt, indicated by different colors in the bars for certain HBV DNA positions.

Samples from 3TC and gRNA/Cas9 combination treatment were subjected to nuclear fractionation followed by Hirt extraction and ExoI+III digestion to obtained enriched cccDNA samples that were analyzed by on-target amplicon sequencing (Fig. 7B). RNA was also extracted from 3TC and gRNA/Cas9 combination treatment and subjected to RNA sequencing (Fig. 7C). PCR and on-target amplicon sequencing of cccDNA-enriched samples after nuclear fractionation followed by Hirt extraction and ExoI+III digestion and RNA-seq showed that the combination of 3TC and gRNA/Cas9 led to the formation of transcriptionally active CRISPRv1 and CRISPRv2 (Fig. S8). The presence of CRISPRv1 after 3TC treatment in combination with gRNA/Cas9 was validated by SB analysis (Fig. 7A). A comparable mutation pattern after gRNA/Cas9 treatment was observed in the absence of 3TC (Fig. S7C and D) in the protocol 2 timeline (RNP treatment at 6 dpi).

Altogether, the results show that the combination of NAs and designer nucleases led to direct cccDNA targeting leading to mutations and generation of different transcriptionally active Cas9-induced variants.

## DISCUSSION

Despite advances in the treatment of chronic HBV infections, lifelong therapies are currently needed (1, 5). Achieving complete HBV cure requires cccDNA elimination from infected hepatocytes. We used CRISPR-Cas9 RNPs to target HBV genome and determine the fate of cccDNA after editing. Several studies suggested cccDNA degradation after gene editing based on the analysis of viral protein released in cell supernatants to assess cccDNA integrity and/or by studying its effect in replication model surrogates, such as plasmids containing HBV genetic information (19–23). HBV plasmids will produce higher-than-physiological HBV DNA copy numbers that could lead to higher-than-biologically relevant numbers of DSBs after Cas9 cleavage. These high numbers of DSBs could be beyond the repair threshold of the cell, which could explain why cccDNA degradation, rather than repair, was observed after Cas9 DSBs. Thus, it is essential to determine the fate of cccDNA after gene editing in models producing authentic cccDNA, such as infected HepG2-NTCP cells (24), and by investigating the effect on cccDNA and its transcripts by qPCR, SB, amplicon sequencing, and RNA sequencing. The main focus of this study was to evaluate if the highly condensed, histone- and viral-protein-associated cccDNA minichromosome is accessible and a direct target for gRNA/Cas9 and to determine the fate of the edited cccDNA.

Gene editing should be fast and precise to limit toxicity and possible off-target effects; therefore, efforts are being made to transiently deliver gRNA/Cas9 as RNPs or mRNA (25, 26). We used RNP lipofection to target HBV DNA. RNP delivery presents several advantages, including fewer off-target mutations, no need of Cas9-expressing stable cell lines, and easy gRNA multiplexing (27). RNPs were also delivered using nanoblades, leading to results similar to those obtained by RNP lipid transfection (Fig. S2A to E). Nanoblade delivery can be used in primary human hepatocytes and liver-humanized mice (14) and may open perspectives to investigate the CRISPR-Cas9 effect in HBV-infected nontransformed hepatocytes.

Previous studies using stable cell lines expressing the gRNA/Cas9 complex or delivery by lentiviruses or plasmid transfection prior to the infection showed a reduction in the viral protein production, attributed to mutations in cccDNA. However, it is possible that incoming virus genome and/or replicative intermediates, in which the gRNA target sequence is present as dsDNA, could be targeted, instead of or equally with the established cccDNA. To show direct cccDNA targeting, we used combinatorial approaches with NA treatment (Fig. 6 and 7). Cells treated with 3TC present a lower replicative intermediate/cccDNA ratio, making cccDNA the main available target for CRISPR-Cas9 cleavage. The reductions in 3.5-kb RNA and viral protein release in 3TC-treated cells suggest that qualitative changes in cccDNA transcription properties play a role in the reduction of the different viral parameters after CRISPR-Cas9 editing. Indeed, it is possible that the reduction in 3.5-kb RNA and viral protein production is an additive effect of different phenomena, i.e., mutations affecting key binding sites for essential transcription factors, DSBs in cccDNA potentially leading to changes in its chromatin state and thus affecting its transcriptional activity, and/or mutations leading to truncated viral proteins.

On-target amplicon sequencing of highly enriched cccDNA after nuclear fractionation, Hirt extraction, and ExoI+III digestion and RNA-seq analysis, in the presence or absence of 3TC, revealed that the most frequent mutations found in the viral RNA were single-nucleotide indels (Fig. 5 and 7). While previous studies showed that single nucleotide deletions were frequent after HBV DNA Cas9 cleavage (17), this is the first study showing that mutated cccDNA is transcriptionally active. Although at lower levels than reads carrying indel mutations, RNA reads that matched transcription of CRISPRv1 were found, indicating that in spite of lacking major transcriptional regulatory regions (28, 29) (enhancer II, basal core promoter, and polyadenylation signal and HBx ORF), this variant was transcriptionally active. HBx is essential for cccDNA transcriptional activity; thus, it is possible that RNP introduction occurs after early expression of HBx (30) or that residual WT cccDNA provides HBx in *trans* to CRISPRv1. Strikingly, the fragment between the Sp5 and Sp7 target sites, containing mainly regulatory regions and the complete HBx ORF, was also repaired, forming the transcriptionally active CRISPRv2 (Fig. 3; Fig. S4 and S5). Thus, it is also possible that HBx is produced from CRISPRv2 and recruited in *trans* to CRIPSRv1, aiding its transcription (31). While these results demonstrate the existence of CRISPR variants after cccDNA editing, it is apparent that the majority of the cccDNA is repaired sequentially after cleavage, leading to indels at the Sp5 and Sp7 target sites. Although it is unclear how these variants are transcribed, previous studies found that naturally occurring mutants lacking pre-C/C regions could produce “pregenomic-like RNA molecules” (32). This was driven from intragenic promoters believed to be inactive in WT genomes (32). Alternatively, mRNAs could be transcribed from the pre-S/S promoter and enhancer I. Deletion of enhancer sequences may downregulate the synthesis of all mRNAs, and the missing poly(A) signal may contribute to production of an mRNA with lower stability.

cccDNA cleavage by Cas9 could generate linear HBV DNA facilitating HBV integration in the host genome. Although the integration rate in these infectious models is expected to be low (33), we analyzed the presence of human-HBV chimeras in the RNA-seq data as a surrogate for viral integration, comparing gRNA/Cas9 treatment versus non-Cas9 treatment controls. The lack of an increased number of chimeras in the presence of gRNA/Cas9 suggests that integration rate is unchanged under these conditions (data not shown). This suggests that transcription of the CRISPR variants from genome integration using cellular promoters is unlikely. However, the possibility that integration may have occurred at different rates in transcriptionally inactive host genomic regions cannot be excluded.

During CHB, integrated HBV DNA can be a source of HBsAg expression, partially responsible for the T-cell exhaustion phenotype (34). Affecting ORF expression from integrated HBV DNA has been considered as a potential treatment approach. However, targeting integrated HBV DNA with Cas9 leads to DSBs in the genomic DNA and genomic instability. If integrated HBV DNA is to be targeted, approaches not leading to DSBs, such as base or prime editing, and Cas9 accessibility to integrated HBV DNA should be explored (35).

Our study showed that CRISPR-Cas9 led to HBV genome damage, resulting in decreased viral replication, and uncovered unexpected aspects of the use of CRISPR-Cas9 to target cccDNA. The use of dual gRNA led to formation of novel transcriptionally active species after CRISPR-Cas9 cleavage. The impact of these variants in host cells and HBV biology may be relevant when studying gene editing in the context of HBV cure research and warrants further investigation.

## MATERIALS AND METHODS

### Cell lines, viral inoculum, and infection conditions.

HBV particles were concentrated from the supernatant of HepAD38 cells (HBV genotype D; kind gift from C. Seeger [Fox Chase Cancer Center, USA]) (36) by filtering and PEG precipitation (37). HepG2-NTCP cells (kind gift from S. Urban [Heidelberg University, Germany]) (38) were seeded at 10^5^ cells/cm^2^ in growth medium (Dulbecco’s modified Eagle medium [DMEM]; high glucose, 1% penicillin-streptomycin, 1% sodium pyruvate, 1% glutamine [Life Technologies], 5% fetal calf serum [HyClone fetal clone II]). From the next day onward, cells were cultured in medium complemented with 2.5% dimethyl sulfoxide (DMSO) (Sigma) as a standard method to increase HBV infection without affecting cell viability (39). After 72 h, cells were infected at a multiplicity of infection of 1,000 (4% PEG, 16 h). For protocol 1, infected cells were replated at 3 dpi at 5 × 10^4^ cells/cm^2^. At 4 dpi, cells were transfected with RNPs, and medium was replaced 24 h posttransfection (hpt). Supernatants and cells lysates were collected at 7 dpi (4 dpt) or 14 dpi (10 dpt). When indicated, cells were treated with 3TC (10 μM) from 4 dpi until cell collection. In protocol 1, for the “rebound control with or without (+/−) 3TC” condition, 3TC was present for 3 days and then removed for a week. For protocol 2, 3TC was added at 4 dpi and cells were replated at 6 dpi at 8 × 10^4^ cells/cm^2^ in the continuous presence or absence of 3TC.

### gRNA design, RNP assembly, and transfection.

The CRISPR RNA (crRNA) sequences for Sp1 to Sp8 were adapted from published studies (40–42), while Sp9 to Sp12 gRNA sequences were designed using the online Benchling platform minimizing homology to the human genome. crRNAs, Neg1 and -2, *trans*-activating crRNA (tracrRNA)-ATTO550, and Streptococcus pyogenes (S.py) Cas9 protein (WT and dead) were purchased from IDT-DNA. gRNAs were *in vitro* assembled by mixing equimolar concentrations of crRNA and tracrRNA (with or without ATTO550, as indicated) at a final concentration of 30 nM, with heating and cooling down at room temperature to allow formation of heteroduplexes. JetCRISPR (PolyPlus transfection) was used for forward transfection in infected HepG2-NTCP cells.

### Real-time PCRs.

RNA and DNA were extracted using the Macherey-Nagel NucleoSpin RNA kit and the Epicentre MasterPure kit, respectively. Real-time PCRs were performed as previously described (43). The 3.5-kb RNA was normalized to the housekeeping gene beta-glucuronidase (GUSB) (Hs99999908_m1; Thermo Fisher Scientific). Total HBV DNA was measured using TaqMan assay Pa03453406_s1 (Life Technologies). For cccDNA quantification, incomplete double-stranded circular DNA was degraded by ExoI+III treatment, and primers targeting a specific region in cccDNA were used (44) (forward, 5′CCGTGTGCACTTCGCTTCA3′; reverse, 5′GCACAGCTTGGAGGCTTGA3′; probe, 5′[6FAM]CATGGAGACCACCGTGAACGCCC[BBQ]). Serial dilutions of an HBV plasmid served as an external quantification standard. Total DNA or cccDNA was normalized to human beta globin (HBB) (Hs00758889_s1; Thermo Fisher Scientific).

### ELISAs for viral antigens.

Enzyme-linked immunosorbent assays (ELISAs) for HBeAg and HBsAg detection in cell supernatants were performed according to the manufacturer’s protocol using the chemiluminescence immunoassay (CLIA) kit from Autobio Diagnostic. All measurements were performed after 3 days of accumulation in medium.

### Southern blotting.

Cells (2 × 10^7^) were processed using a modified Hirt extraction followed by ExoI+III digestion (45). Samples were then quantified by Qubit, and ND2 qPCR was used to normalize the seeding amount to mitochondrial DNA. Samples were run in a 1.2% agarose gel for 16 h at 15 V. Samples in the agarose gel were depurinated using HCl followed by NaOH treatment and neutralization before transfer to a nylon membrane using a Whatman Turbo blotter. Membranes were blocked and incubated with branched-DNA probes to detect the negative-strand HBV DNA with modifications of the protocol described in reference 46 or with digoxigenin (DIG) DNA probes as described in reference 47.

### RNA sequencing.

RNA was extracted using TRIzol followed by Turbo I DNase treatment. An RNA integrity number of >9.6 was confirmed with a Bioanalyzer prior to submission to Genewiz. cDNA libraries were constructed using a NEBNext Ultra RNA library preparation kit with rRNA depletion. Paired-end sequencing was conducted using Illumina MiSeq with a read length of 150 bp (average of ∼100 × 10^6^ reads/sample). Sequencing reads were aligned to the human and HBV reference genome (GenBank no. U95551.1) using STAR (48). To calculate the frequency, two variant callers (Pindel and HaplotypeCaller) were used to determine the number of reads supporting a given mutation. The ratio of mutations to the total number of reads (mutated or not) was then manually calculated using the data provided by the variant callers. For the CRISPR variants, custom reference genomes were generated based on the expected cleavage sites of S.py Cas9 (−3 from the PAM) followed by recircularization of each double-cleaved fragment. Coverage calculation per position was performed using GenomeCov from the toolkit bedtools. The original coverage per position in the genome ranged from 2,000 to 15,432.

### Amplicon sequencing.

Nuclear extraction of 6 × 10^6^ cells was performed in cell lysis buffer (5 mM PIPES, 85 mM KCl, 0.5% NP-40, 1 mM phenylmethylsulfonyl fluoride [PMSF], 1× protease inhibitor cocktail [PIC; Thermo Fisher]). After “tight” treatment with Dounce and centrifugation (10 min, 4,500 rpm), nuclei were resuspended in 1× Tris-EDTA (TE) buffer. Episomal DNA was enriched using a modified Hirt extraction (45), followed by phenol-chloroform DNA purification, ExoI+III digestion, and Zymo column cleanup. Primers pairs for nested PCR using HiFi Q5 polymerase were designed to flank Sp5 and Sp7 target sites for on-target amplicon sequencing. To calculate the frequency, two variant callers (Pindel and HaplotypeCaller) were used to determine the number of reads supporting a given mutation. The ratio of mutations to the total number of reads (mutated or not) was then manually calculated using the data provided by the variant callers. For the CRISPR variants, same primers were combined in different pairs around the novel predicted junctions. Amplicons of around 300 bp were then submitted for next-generation sequencing (NGS) to Genewiz facilities (Amplicon-EZ) (Fig. S8E).

### Statistical analysis.

Statistical analysis was performed using Prism 7 software (GraphPad Software, San Diego, CA, USA). The Kruskal-Wallis test and Dunn’s multiple-comparison test were used to compare numerical data.

### Data availability.

DNA amplicon-sequencing and RNA sequencing data are be available at the European Nucleotide Archive (ENA) under accession number PRJEB51625.
