# Supplementary figures and images for: CRISPR-Cas9 Targeting of Hepatitis B Virus Covalently Closed Circular DNA Generates Transcriptionally Active Episomal Variants
Source: mBio. 2022 Apr 7;13(2):e02888-21. doi: 10.1128/mbio.02888-21 (PMC9040760; doi:10.1128/mbio.02888-21)

Supplementary Figure 1

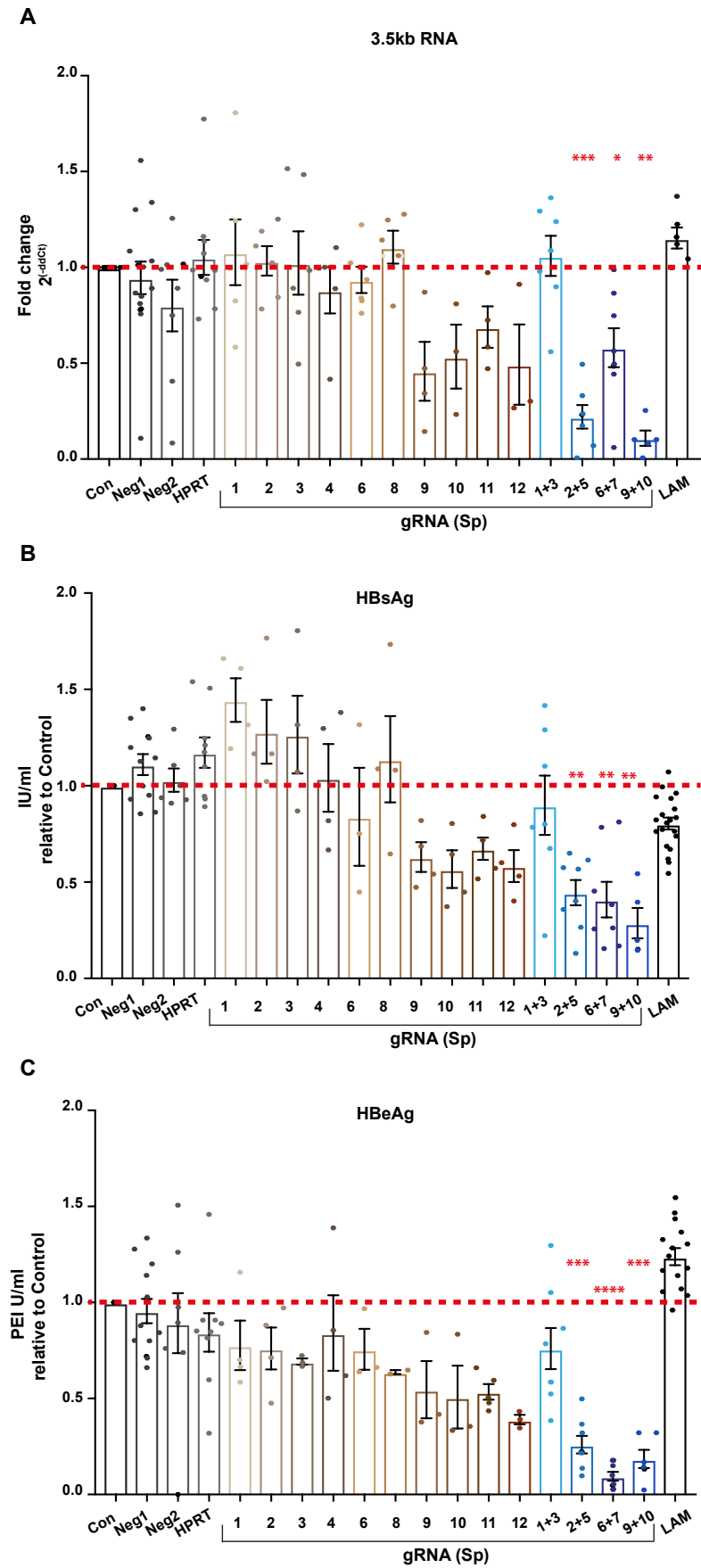

Supplement: FIG S1 [file mbio.02888-21-sf001.pdf]

Supplementary Figure 2

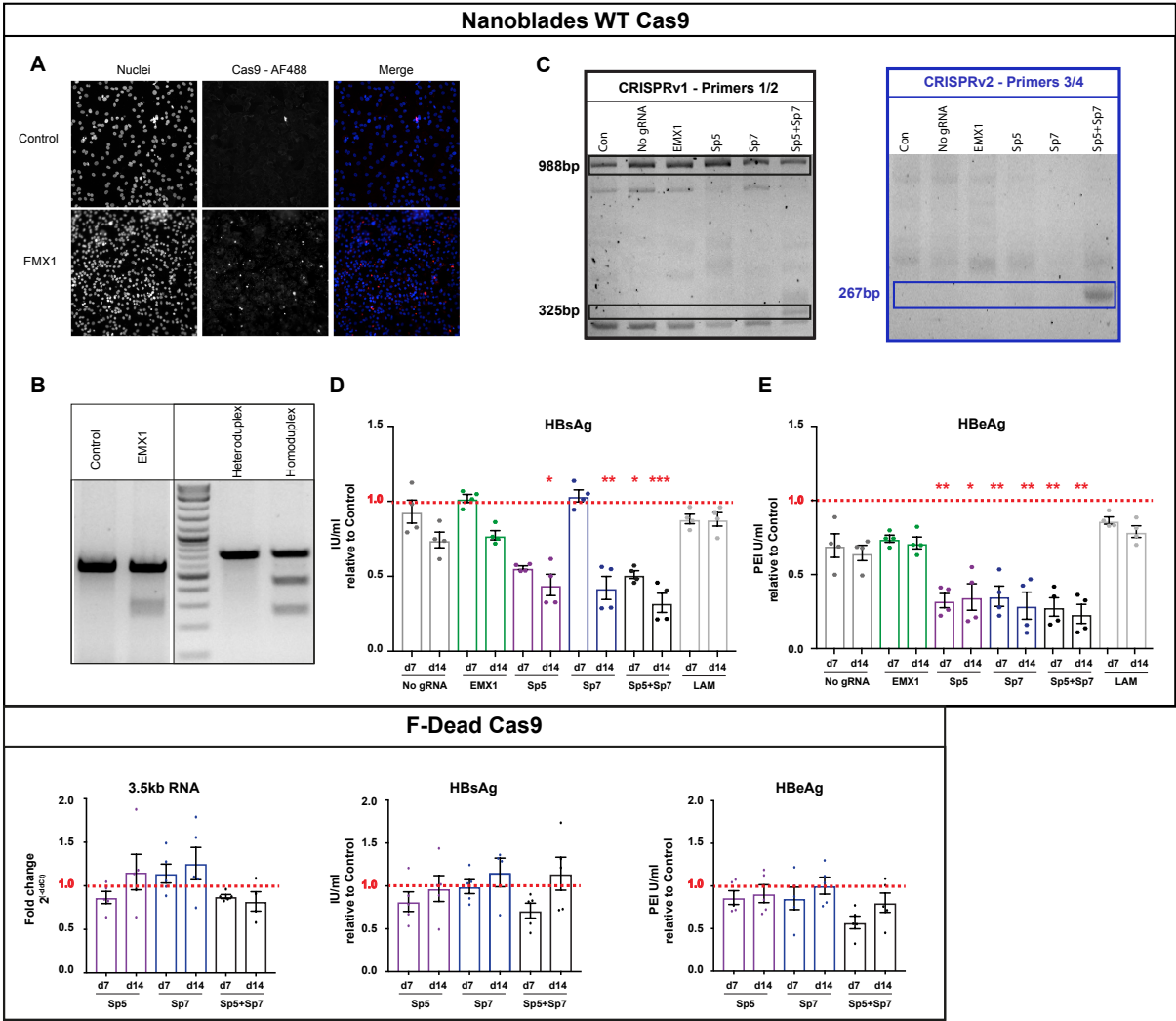

Supplement: FIG S2 [file mbio.02888-21-sf002.pdf]

Supplementary Figure 3

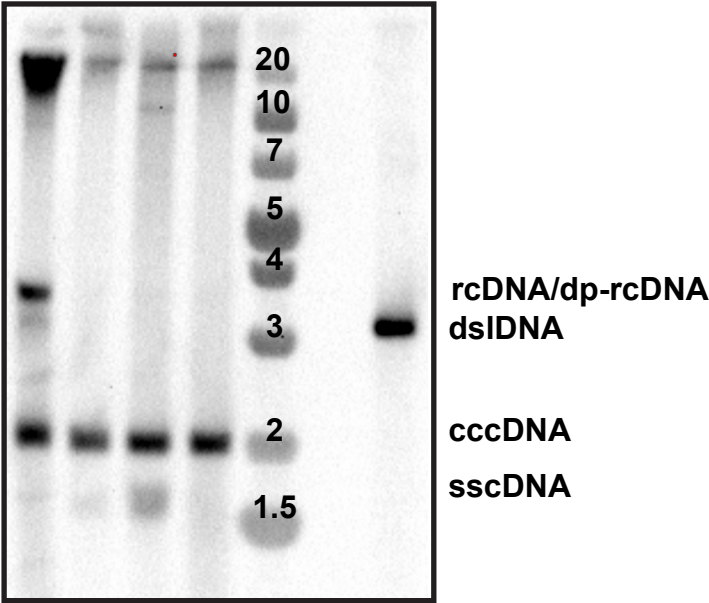

|          |  |   |   |   |             |            |
|----------|--|---|---|---|-------------|------------|
| Exol+III |  | X | X |   | 1kb+ ladder | Linear HBV |
| T5       |  |   |   | X |             |            |
| 95C 5min |  |   | X |   |             |            |

Supplement: FIG S3 [file mbio.02888-21-sf003.pdf]

Supplementary Figure 6

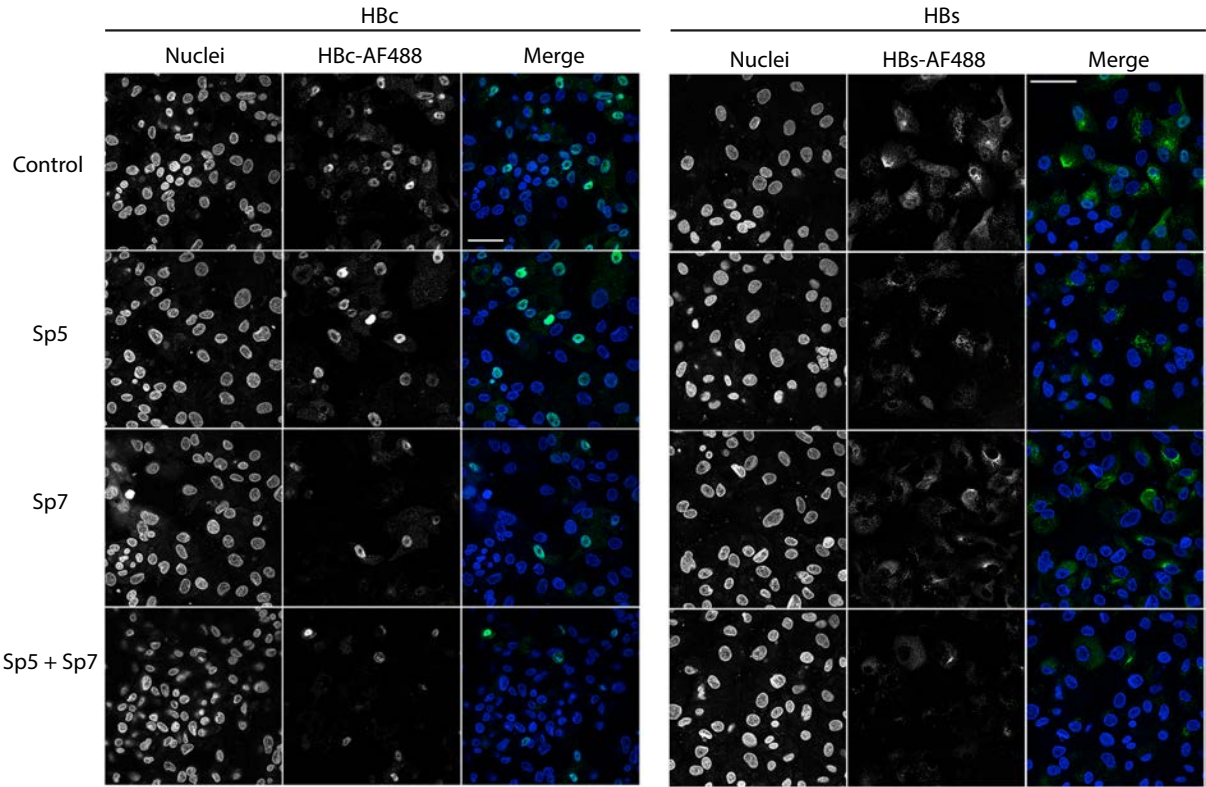

Supplement: FIG S6 [file mbio.02888-21-sf006.pdf]

Supplementary Figure 7

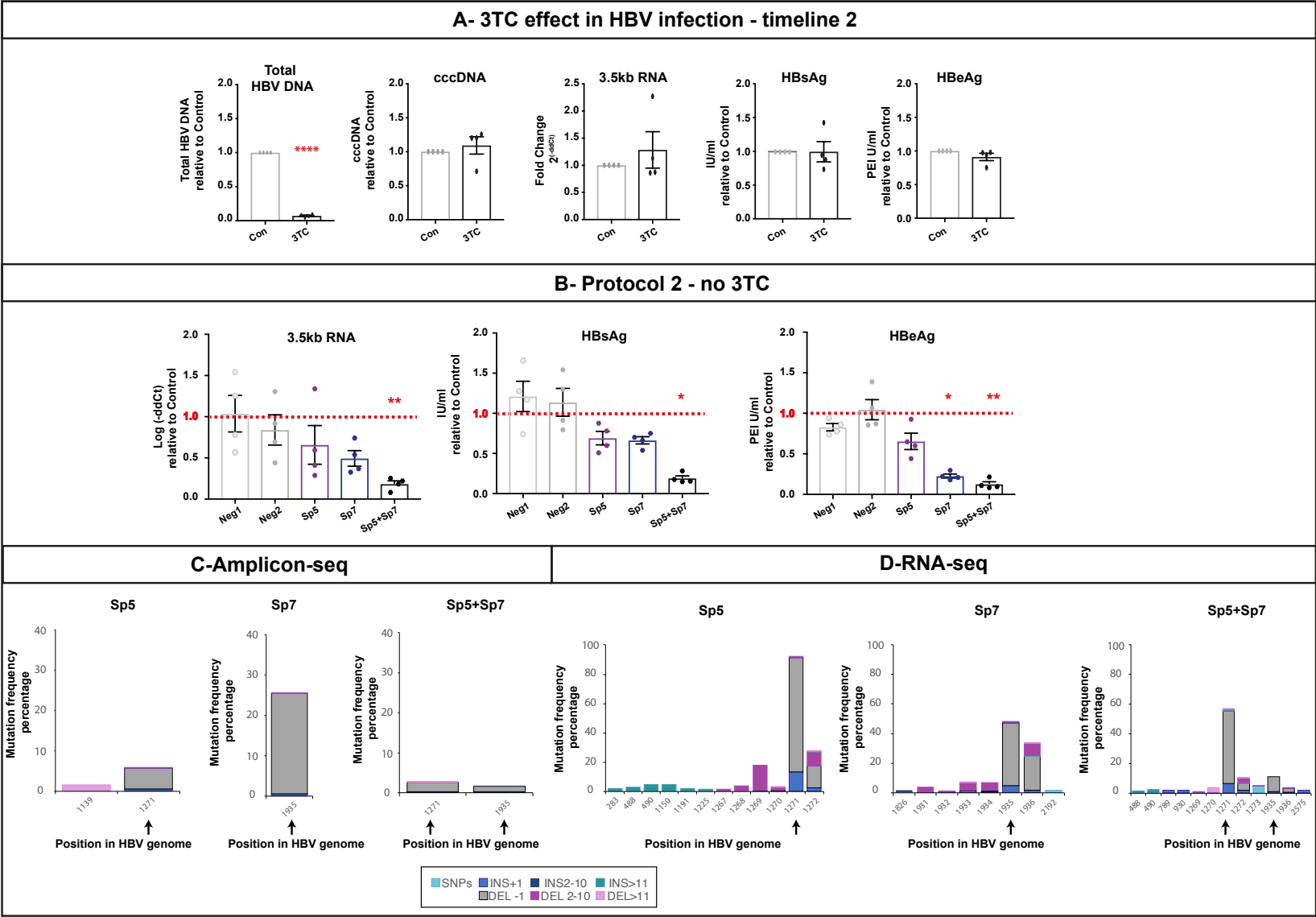

Supplement: FIG S7 [file mbio.02888-21-sf007.pdf]
