# Supplementary material for: CRISPR-Cas9 Targeting of Hepatitis B Virus Covalently Closed Circular DNA Generates Transcriptionally Active Episomal Variants
Source: mBio. 2022 Apr 7;13(2):e02888-21. doi: 10.1128/mbio.02888-21 (PMC9040760; doi:10.1128/mbio.02888-21)

Supplementary Figure 8

A

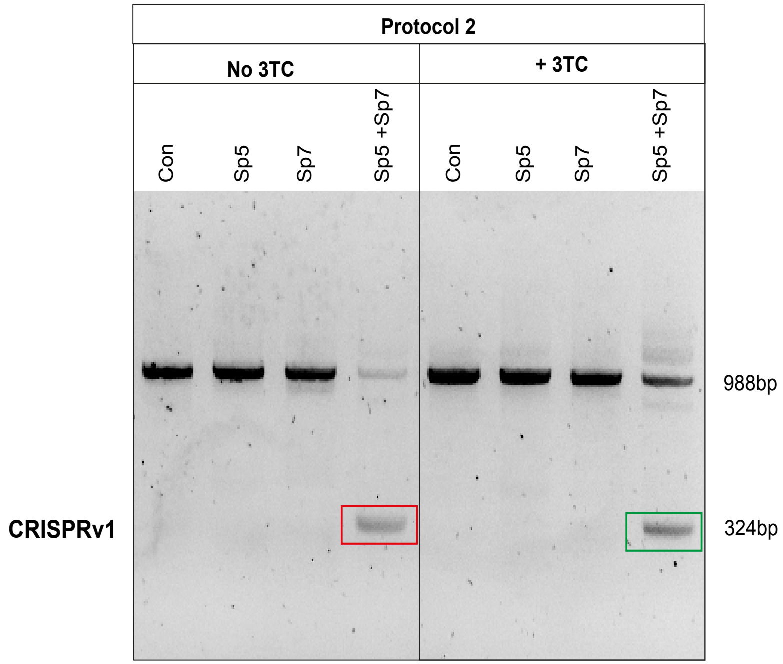

B

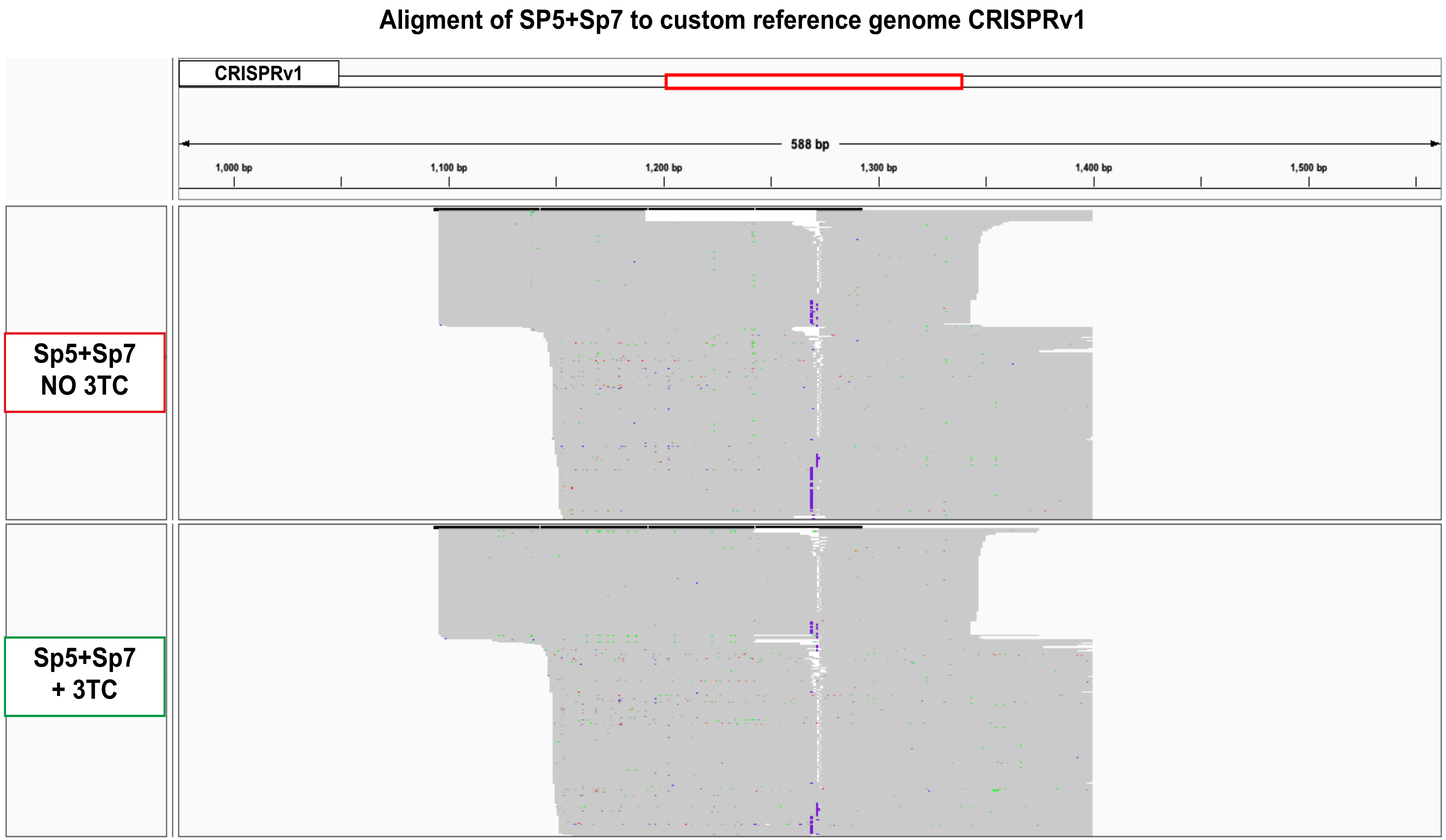
Aligment of SP5+Sp7 to custom reference genome CRISPRv1
CRISPRv1
588 bp
1,000 bp
1,100 bp
1,200 bp
1,300 bp
1,400 bp
1,500 bp
Sp5+Sp7
NO 3TC
Sp5+Sp7
+ 3TC

C

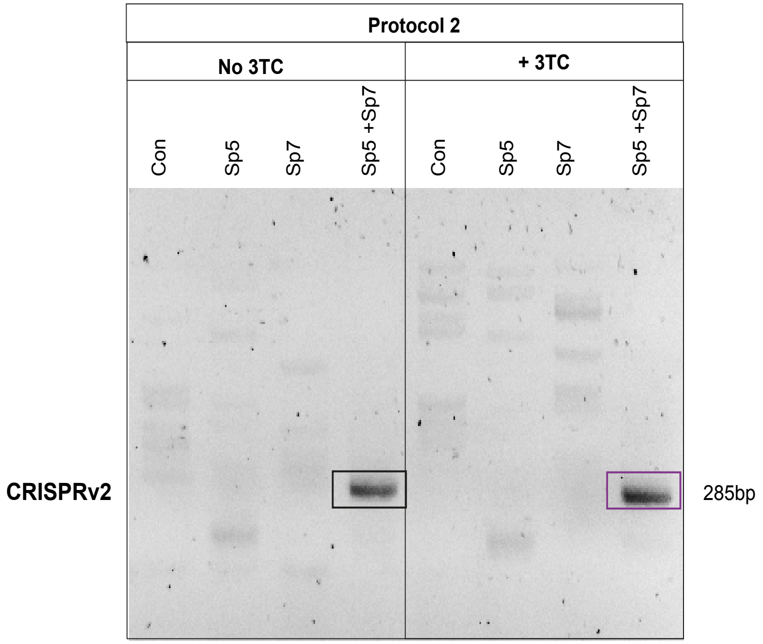

Protocol 2
No 3TC
+ 3TC
Con
Sp5
Sp7
Sp5 +Sp7
Con
Sp5
Sp7
Sp5 +Sp7
CRISPRv2
285bp

D

Aligment of SP5+Sp7 to custom reference genome CRISPRv2

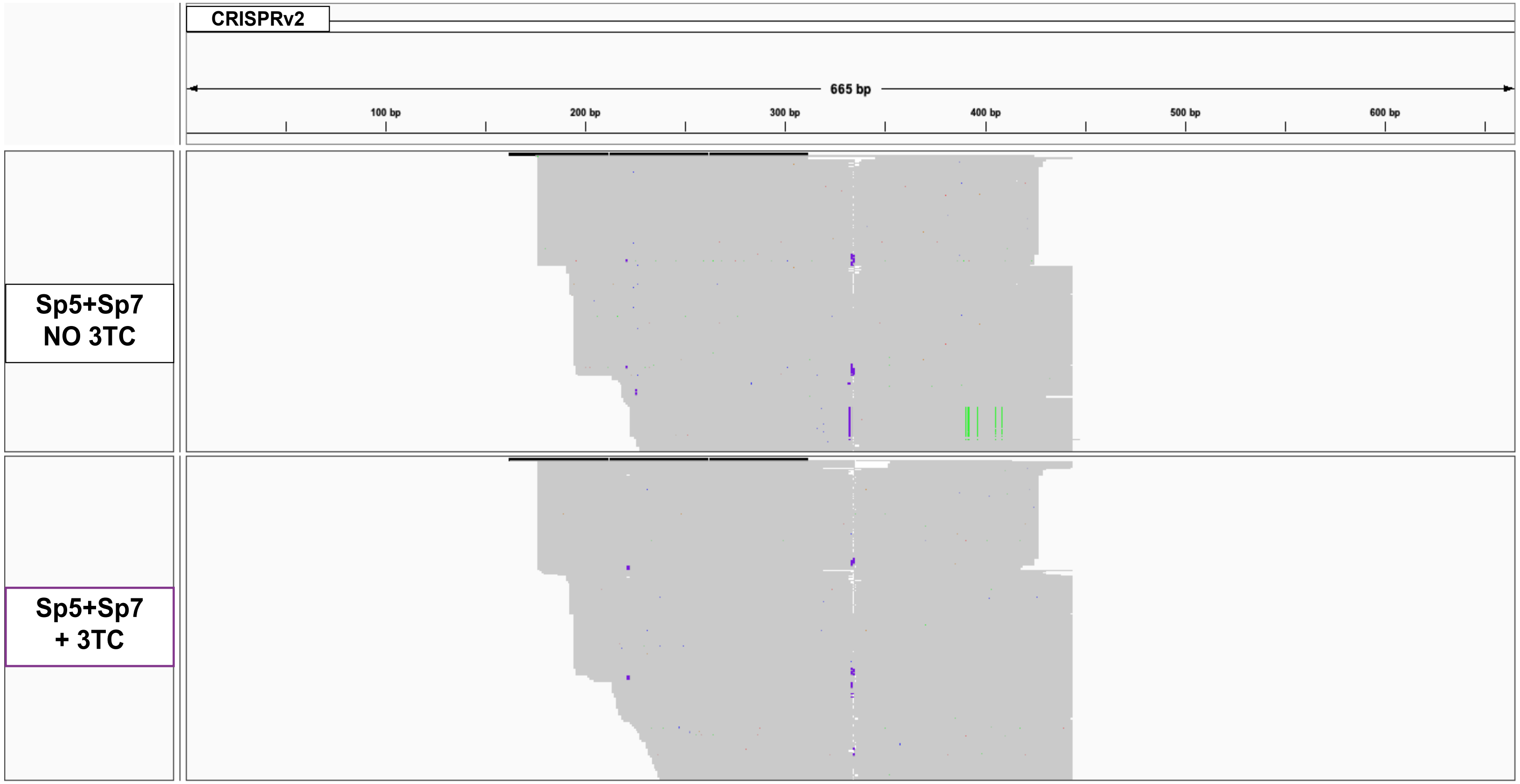

E

PCR1							
**Starting material**	**Amplicon target**	**Pair**	**Primer name**	**Primer sequence**	**Ta***	**Ct**	**size**
5 ng of cccDNA-enriched by Hirt protocol	CRISPRv1	A_1-2	Primer 1	CTTTCTCGCCAACTTACAAGG	64°C	35	988 (WT)-324 (CRISPRv1)
			Primer 2	AAAGAATTGCTTGCCTGAGTG			
	CRISPRv2	B_4-3	Primer 3ext	GGTCTTTGTACTAGGAGGCTGTAG	65°C	35	285#
			Primer 4	TAGCAGCCATGGATACGATG			
	Sp5 target	1-4	Primer 1	CTTTCTCGCCAACTTACAAGG	64°C	35	290
			Primer 4	TAGCAGCCATGGATACGATG			
	Sp7 Target	3-2	Primer 3	GAGGCTGTAGGCATAAATTGG	64°C	35	305
			Primer 4	AAAGAATTGCTTGCCTGAGTG			
**PCR2**							
**Starting material (for 4x25µl of A reaction)**		**Pair**	**Primer name**	**Primer sequence**	**Ta***	**Ct**	**size**
5% of PCR A	CRISPRv1	C_1-2bis	Primer 1	CTTTCTCGCCAACTTACAAGG	64°C	32-35	304#
			Primer 2ext	GCAGTATGGTGAGGTGAACAATG			
5% of PCR B	CRISPRv2	D_4-3bis	Primer 3	GAGGCTGTAGGCATAAATTGG	64°C	32-35	267#
			Primer 4ext	AGCCATGGATACGATGTATATTTGC			
20 ng of PCR E DNA	Sp5 target	1-4	Primer 1	CTTTCTCGCCAACTTACAAGG	64°C	25	290
			Primer 4	TAGCAGCCATGGATACGATG			
20 ng of PCR F DNA	Sp7 Target	3-2	Primer 3	GAGGCTGTAGGCATAAATTGG	64°C	25	305
			Primer 4	AAAGAATTGCTTGCCTGAGTG			

F

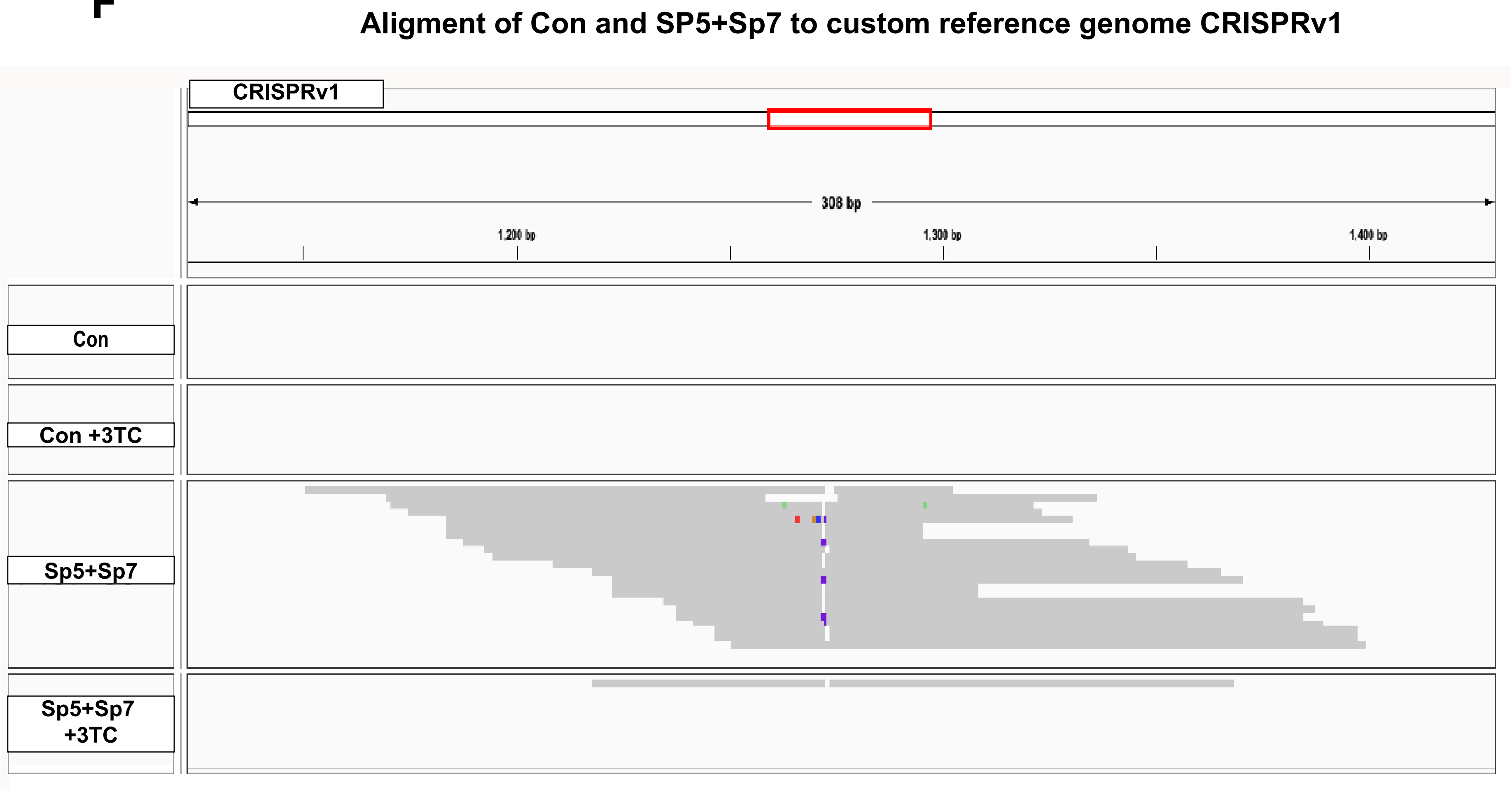
Aligment of Con and SP5+Sp7 to custom reference genome CRISPRv1
CRISPRv1
308 bp
1,200 bp
1,300 bp
1,400 bp
Con
Con +3TC
Sp5+Sp7
Sp5+Sp7
+3TC

G

Aligment of Con and SP5+Sp7 to custom reference genome CRISPRv2

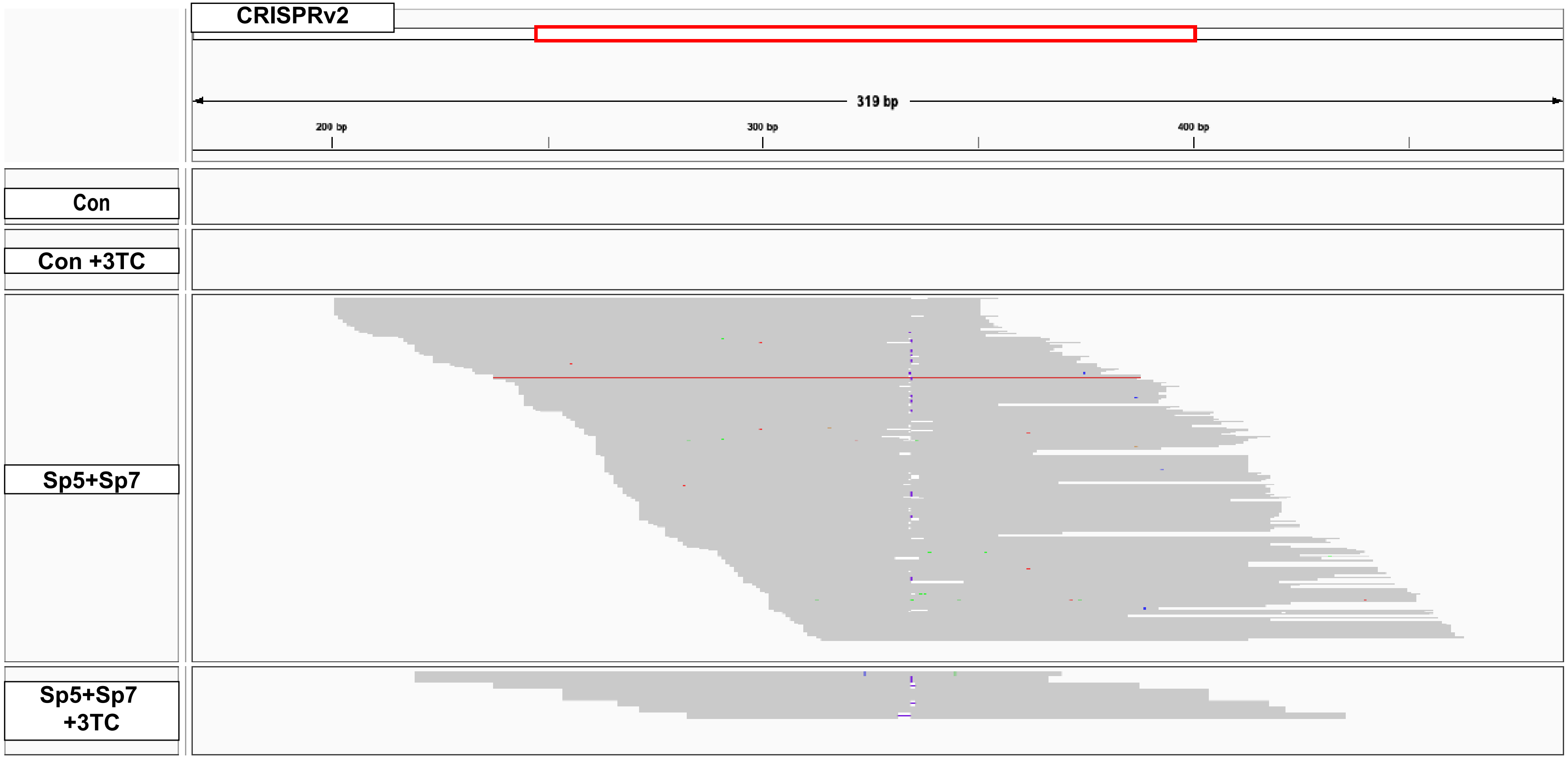

Supplement: FIG S8 [file mbio.02888-21-sf008.pdf]
