# Supplementary material for: CRISPR-Cas9 Targeting of Hepatitis B Virus Covalently Closed Circular DNA Generates Transcriptionally Active Episomal Variants
Source: mBio. 2022 Apr 7;13(2):e02888-21. doi: 10.1128/mbio.02888-21 (PMC9040760; doi:10.1128/mbio.02888-21)
Supplement: TEXT S1 [file mbio.02888-21-s0001.docx]

**CRISPR-Cas9 targeting of Hepatitis B virus cccDNA generates transcriptionally active episomal variants**

Martinez Maria Guadalupe, Combe Emmanuel, Inchauspe Aurore, Mangeot Phillipe Emmanuel, Delberghe Elodie, Chapus Fleur, Neveu Gregory, Alam Antoine, Carter Kara, Testoni Barbara, Zoulim Fabien

**Supplementary Methods**

*Nanoblade production and delivery in HepG2-NTCP cells.* Nanoblades were produced following the protocol described in[2]. Briefly, gesicle producing 293T cells were transfected with plasmids encoding GagMLV-Cas9, Gag-POLMLV, gRNA expressing plasmid(s), VSV-G and BaEVRless and supernatants were collected after 40h. Nanoblade containing medium was clarified and filtered through a 0.8um pore-size filter before ultracentrifugation. Pellet was resuspended in PBS1X. Nanoblades batches were dosed by nitrocellulose blot and concentrations of Cas9 was measured at around 2µM. Following protocol 1, infected cells were replated at 3dpi and transduced with nanoblades assembled with no gRNA, gRNA targeting the cellular control EMX-1, Sp5, Sp7 or the combination Sp5+Sp7 (10pmol Cas9 per well in a 48-w plate) at 4dpi. Medium was replaced 24 hpt for medium complemented with 2.5% DMSO. Supernatants were collected at 7dpi (4dpt) or 14dpi (10dpt) and cell lysates were collected at 14dpi.

**Supplementary Results**

*Effect of single gRNA treatment targeting different HBV genomic regions*

Sp5 crRNA sequence targets the HBV DNA sequence that codifies for Pol and the transcription start site (TSS)[3] of HBx while SP7 targets both early HBe/HBc. While Sp5 in singleplex does not lead to sustained decrease of viral protein production (Fig.2 B-C), Sp7 targeting early HBe/HBc sequence lead to an expected decrease in HBe secretion (Fig.2C, 6C) and intracellular HBcAg (Fig.S10). Sp9-Sp12, all targeting regulatory regions in the HBV genome, lead to an important decrease of 3.5kb RNA, HBeAg and HBsAg (Fig.S1), suggesting that mutations induced by these gRNAs could affect sites for binding of moieties essential for cccDNA transcription and RNA processing. While several gRNAs generated similar decreases in HBV 3.5kb RNA, they did not have as large an effect in HBe secretion as did Sp7. This difference could be explained by the target sequence of Sp7 being the only one directly affecting HBe/HBc and/or editing efficiency due to the crRNA sequence.

*Effect of gRNA/Cas9 targeting in intracellular viral protein production*

The lead mutation after Sp5/Cas9 targeting is a single nucleotide deletion in position 1271 of the HBV genome, which leads to mutations both in HBx promoter and a change in the open reading frame (ORF) of the polymerase resulting in an early termination (STOP codon in the position 1284, instead of 1623 leading to a shorter polymerase protein (719aa instead of 832aa for the wild-type). In the case of Sp7/Cas9 treatment its more frequent mutation is a single nucleotide deletion in position 1935 of the HBV genome that changes the HBc/HBe ORF leading to an early stop codon (position 1992 instead of 2451, translating into a 59aa protein instead of 212aa). Immunofluorescence studies were performed to assess how these mutations affect HBs and HBc protein expression and localization (Fig.S6). Decrease in the protein expression, but not obvious re-localization of both proteins was found to be especially efficient when the combination of Sp5 and Sp7 was used, which agrees with the reduction of HBe and HBs protein secretion and reduction of 3.5kb HBV RNA found in these conditions by ELISA and qPCR (Fig.2).

**Supplementary References**

[1] Testoni B, Durantel D, Lebossé F, Fresquet J, Helle F, Negro F, et al. Ribavirin restores IFNα responsiveness in HCV-infected livers by epigenetic remodelling at interferon stimulated genes. Gut 2016;65:672–82. https://doi.org/10.1136/gutjnl-2014-309011.

[2] Mangeot PE, Risson V, Fusil F, Marnef A, Laurent E, Blin J, et al. Genome editing in primary cells and in vivo using viral-derived Nanoblades loaded with Cas9-sgRNA ribonucleoproteins. Nat Commun 2019;10:45. https://doi.org/10.1038/s41467-018-07845-z.

[3] Altinel K, Hashimoto K, Wei Y, Neuveut C, Gupta I, Suzuki AM, et al. Single-Nucleotide Resolution Mapping of Hepatitis B Virus Promoters in Infected Human Livers and Hepatocellular Carcinoma. J Virol 2016;90:10811–22. https://doi.org/10.1128/JVI.01625-16.

[4] Thyme SB, Akhmetova L, Montague TG, Valen E, Schier AF. Internal guide RNA interactions interfere with Cas9-mediated cleavage. Nat Commun 2016;7:11750. https://doi.org/10.1038/ncomms11750.
