# Supplementary material for: CRISPR-Cas9 Targeting of Hepatitis B Virus Covalently Closed Circular DNA Generates Transcriptionally Active Episomal Variants
Source: mBio. 2022 Apr 7;13(2):e02888-21. doi: 10.1128/mbio.02888-21 (PMC9040760; doi:10.1128/mbio.02888-21)

Supplementary Figure 5

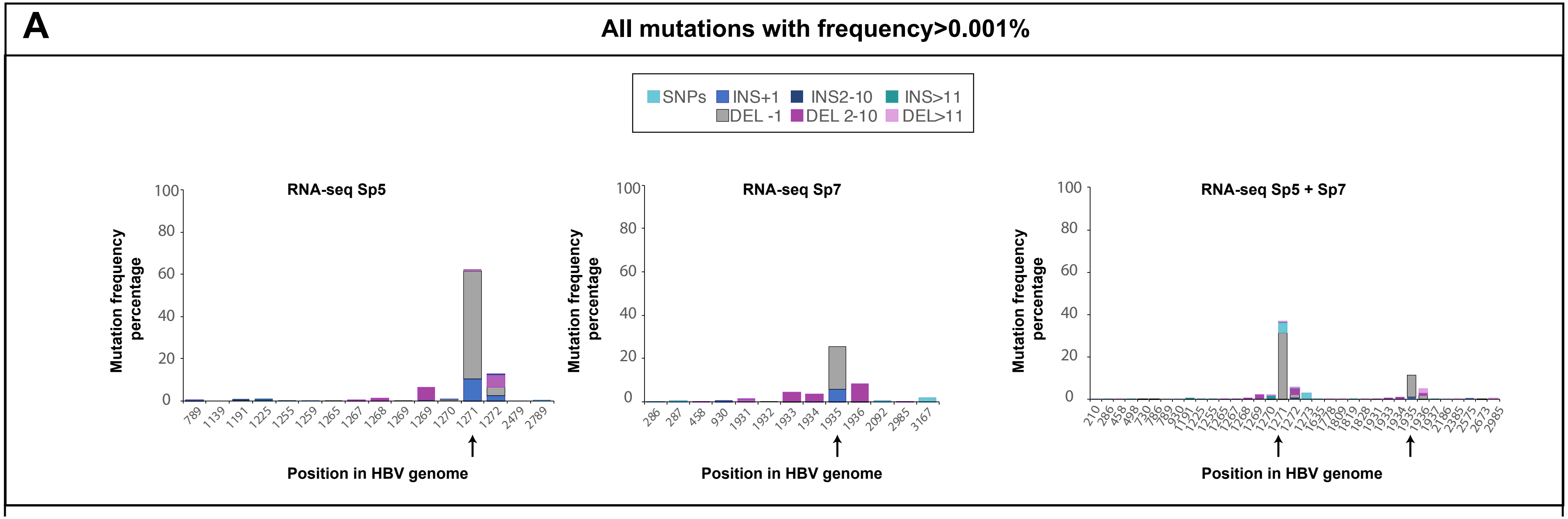

B **Aligment of Con and SP5+Sp7 to custom reference genome CRISPRv1**

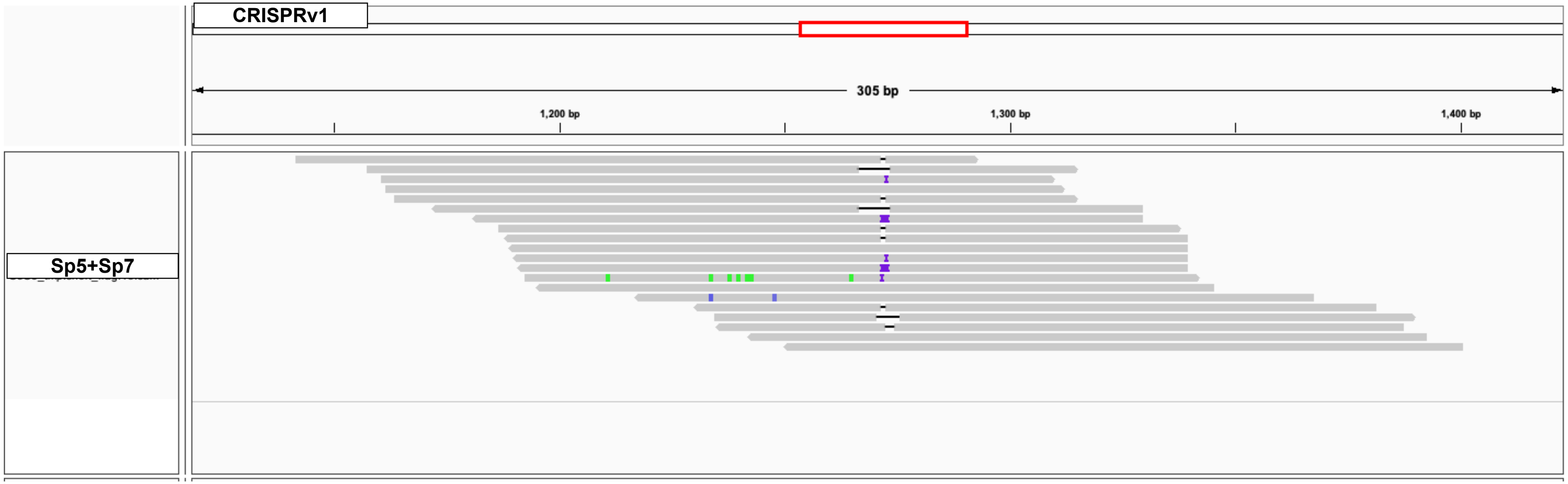

C

Aligment of Con and SP5+Sp7 to custom reference genome CRISPRv2

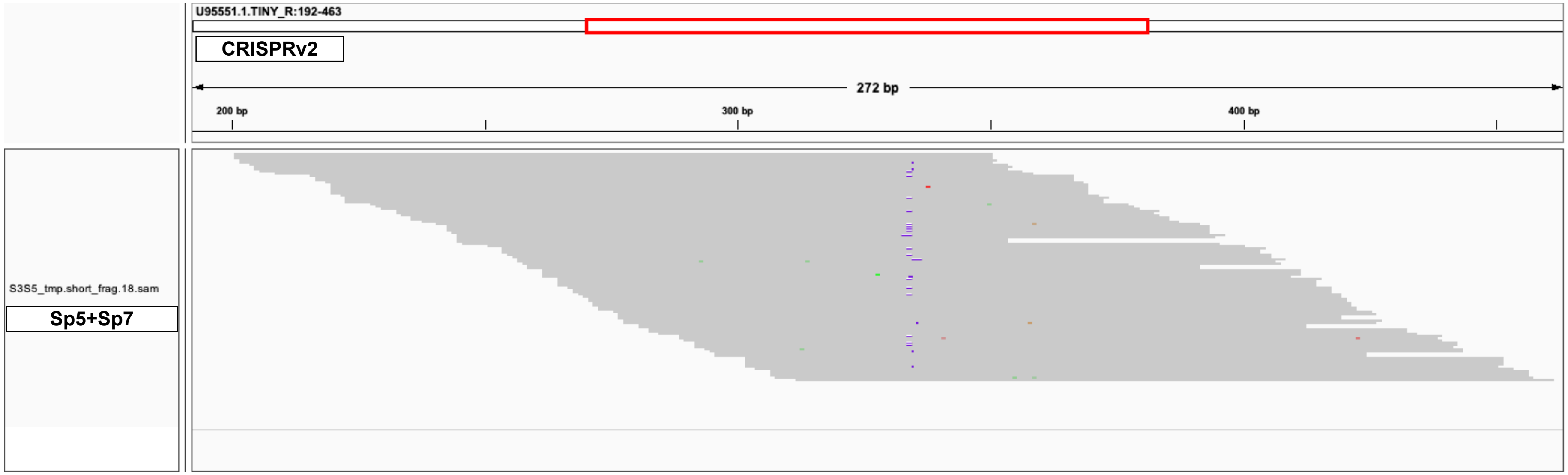

Supplement: FIG S5 [file mbio.02888-21-sf005.pdf]
